# Global fibroblast activation throughout the left ventricle but localized fibrosis after myocardial infarction

**DOI:** 10.1038/s41598-017-09790-1

**Published:** 2017-09-07

**Authors:** Chandan K. Nagaraju, Eef Dries, Natasa Popovic, Abhishek A. Singh, Peter Haemers, H. Llewelyn Roderick, Piet Claus, Karin R. Sipido, Ronald B. Driesen

**Affiliations:** 1 Experimental Cardiology, Department of Cardiovascular Sciences, KU Leuven, Herestraat 49, Leuven, 3000 Belgium; 2 Cardiovascular Imaging and Dynamics, Department of Cardiovascular Sciences, KU Leuven, Herestraat 49, Leuven, 3000 Belgium

**Keywords:** Mechanisms of disease, Cardiovascular diseases

## Abstract

Fibroblast (Fb) differentiation and interstitial fibrosis contribute to cardiac remodeling and loss of function after myocardial infarction (MI). We investigated regional presence and regulation of fibrosis in a pig MI model. *In vivo* analysis of regional function and perfusion defined three regions: the scar, the myocardium adjacent to the scar (MI_adjacent_, reduced function, reduced perfusion reserve), and the remote myocardium (MI_remote_, minimal functional deficit, maintained perfusion). Interstitial and perivascular fibrosis, and increase of collagen type I, was only observed in the MI_adjacent_. Fb activated protein-alpha (FAP-α) was enriched in MI_adjacent_ compared to MI_remote_. TGF-β1, which triggers Fb differentiation, was upregulated in both MI_adjacent_ and MI_remote_, whereas lysyl oxidase, a regulator of collagen cross-linking, and the proteoglycans decorin and biglycan were only increased in the MI_adjacent_. Fb isolated and cultured for 4 days had myoFb characteristics with little difference between MI_remote_ and MI_adjacent_, although RNA sequencing revealed differences in gene expression profiles. Fbs from all regions maintained proliferative capacity, and induced contraction of 3-D collagen matrices but scar myoFb was more effective. These data suggest that after MI, signaling through TGF-β1, possibly related to increased mechanical load, drives Fb activation throughout the left ventricle while regional signaling determines further maturation and extracellular matrix remodeling after MI.

## Introduction

Fibroblasts (Fb) belong to the most abundant cell type in the heart next to myocytes and endothelial cells^1^. They are an integral part of the heart and contribute to the cardiac structure and function during development, normal function and cardiac dysfunction^2–4^. Fb have different developmental origin with phenotype variability though most are derived from pro-epicardium^5^. Fb derived from a healthy ventricle can be distinguished from their atrial counterparts at a structural and transcriptome level^6^. In the healthy myocardium, Fb are quiescent, lack contractile stress fibers^7^ and have a major role in synthesis and organization of the extracellular matrix (ECM)^8, 9^. Fb are sensitive to mechanical and chemical alterations, which develop during the progression of heart disease^10^. Several systemic and local factors such as angiotensin II and transforming growth factor β1 (TGF- β1) trigger the phenotypical transition of resident Fb^11, 12^. Initially, Fb differentiate to proto-myofibroblasts defined as an intermediate phenotype containing stress fibers lacking α-smooth muscle actin (α-SMA)^7^. Further differentiation into myofibroblasts (MyoFb) is associated with the development of a mature α-SMA stress fiber network. At this stage, MyoFb have acquired the capacity to remodel the ECM by exerting high intracellular force via activated integrins^13^ and cadherin receptor proteins^14^. In addition, they produce high levels of pro-collagen fibrils, which after secretion are cross-linked by lysyl oxidase (LOX) into mature collagen fibers^15, 16^. Persistent activation and differentiation of Fb drives further maturation of the compact scar tissue. After myocardial infarction (MI), the scar replaces lost cardiac myocytes and will aid in preserving the structural and functional integrity of the left ventricle (LV)^17, 18^. Unlike dermal scar, which is acellular, cardiac scars are composed of living MyoFb embedded in cross-linked collagen^19^. These MyoFb can eventually achieve a senescent state in which they persist in the scar for years^20^. However, in the non-infarcted myocardium, differentiated Fb contribute to reactive fibrosis, which can classified as either interstitial, diffuse, patchy or perivascular fibrosis^21^. Interstitial fibrosis will alter myocardial compliance promote hypertrophy and diastolic dysfunction^22, 23^. Apart from ventricular dilation, fibrosis also promotes slippage of cardiac myocytes^24, 25^. The origin and signaling pathways that direct MyoFb to the injury site after MI are not fully unraveled^26, 27^. Most recent data indicate that in pressure overload MyoFb arise from activation of resident Fb^28^. MyoFb can also result from endothelial to mesenchymal transition of resident endothelial cells^29, 30^.

Region-specific fibroblast phenotypes in the scar tissue and in non-infarcted myocardium and their functional role during post-MI remodeling are less studied. Rodents, the most used animal model, typically have a large MI with profound reduction of LV function and activation of circulating hormones that influence global remodeling. In the pig model of ischemic cardiomyopathy, previously developed in our laboratory, MI induced by severe coronary stenosis, impacts on LV function, reducing ejection fraction (EF) from 60% to 40% without however frank heart failure^31, 32^. Based on *in vivo* magnetic resonance imaging (MRI) of structure, function and perfusion, we can distinguish three regions: the scar without contraction and severely limited perfusion (i), the myocardium adjacent (MI_adjacent_) to the scar with reduced function and reduced perfusion reserve (ii), and the remote myocardium (MI_remote_) with minimal functional deficit and maintained perfusion (iii). Wall stress is increased throughout the LV^32^. In the present study, we investigate whether Fb differentiation and function is differentially regulated in these regions and how this impacts on fibrosis. We isolated Fb from different LV regions in MI and SHAM animals and studied their phenotypical and functional characteristics in short term (4 days) culture since *in vitro* culture of Fb for an extended period leads to artificial alterations in their properties^33^. Concomitantly, we analyzed interstitial fibrosis in matched tissue samples and compared activation of signaling pathways.

## Results

### Interstitial and perivascular fibrosis in the adjacent myocardium

**Figure 1:**
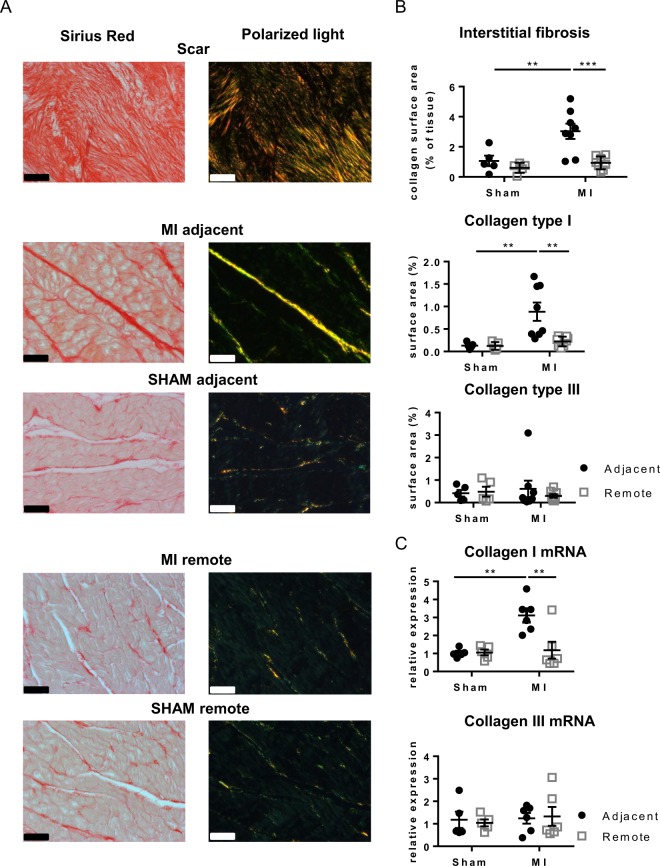
Interstitial fibrosis in different regions of the LV. (**A**) Representative images of Sirius red stained sections and polarized light microscopy from the adjacent, remote and scar tissue of SHAM and MI. (**B**) Analysis of interstitial fibrosis, and in polarized light collagen type I (red-yellow) and collagen type III (green) in SHAM and MI. Scale bars represent 50 µm. (**C**) Collagen type I and III mRNA expression in the adjacent and remote myocardium of SHAM and MI. (**p < 0.01: ***p < 0.001) (2-way ANOVA with Bonferroni post hoc test).

Scar tissue is mainly composed of collagen, with predominant type I fibers and some type III (Fig. 1A, top panels); collagen is organized into highly cross-linked fibers that extend into the neighboring border zone (Supplemental Fig. 1A,B). In MI_adjacent_, there is a 3-fold increase in interstitial fibrosis compared with SHAM (Fig. 1A, middle panels). The increase in interstitial fibrosis is predominantly accumulation of collagen type I fibers (yellow-red color) without apparent changes in collagen type III (green color). Ultra-structural evaluation by transmission electron microscopy suggests the presence of cross-linked collagen type I fibers enveloping the sarcolemma of the cardiac myocytes (Supplemental Fig.1D). In contrast, the MI_remote_ has no interstitial fibrosis, no change in collagen type I and III distribution and absence of cross-linked collagen fibers (Fig. 1A (lower panels); Supplemental Fig. 1F); the data are comparable to the matched region in SHAM. Quantification of interstitial fibrosis is shown in Fig. 1B. mRNA levels confirmed the histological observations showing a 3-fold increase of collagen type I in MI_adjacent_ when compared to SHAM (Fig. 1C), whereas collagen type III in MI revealed no differences (Fig. 1C).

**Figure 2:**
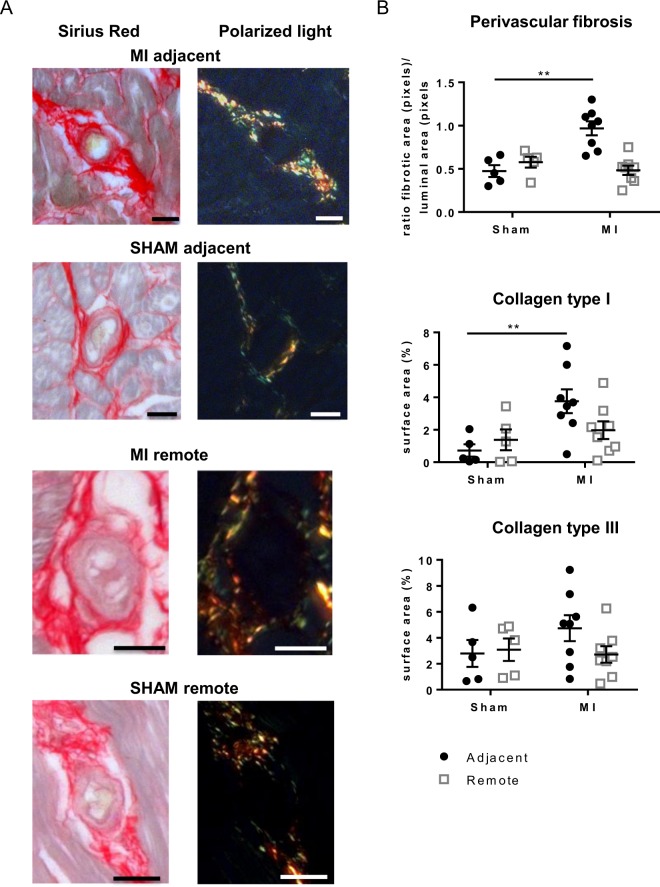
Perivascular fibrosis in the adjacent myocardium. (**A**) Representative images of Sirius red stained arterioles from the adjacent and remote myocardium of SHAM and MI acquired via light and polarization microscopy. (**B**) Quantification of perivascular fibrosis and collagen isoforms within the perivascular area. Scale bars represent 20 µm. (**p < 0.01) (2-way ANOVA with Bonferroni post hoc test).

Concomitantly with interstitial fibrosis, there is on average a 2-fold increase in perivascular fibrosis around arterioles in MI_adjacent_ as compared to SHAM (Fig. 2A,B). Moreover, the perivascular area in the MI_adjacent_ has on average a 5-fold increase in collagen type I, whereas collagen type III remains unchanged (Fig. 2A,B). The MI_remote_ has no increase of perivascular fibrosis compared to SHAM and shows no changes in collagen type I and III (Fig. 2A,B).

**Figure 3:**
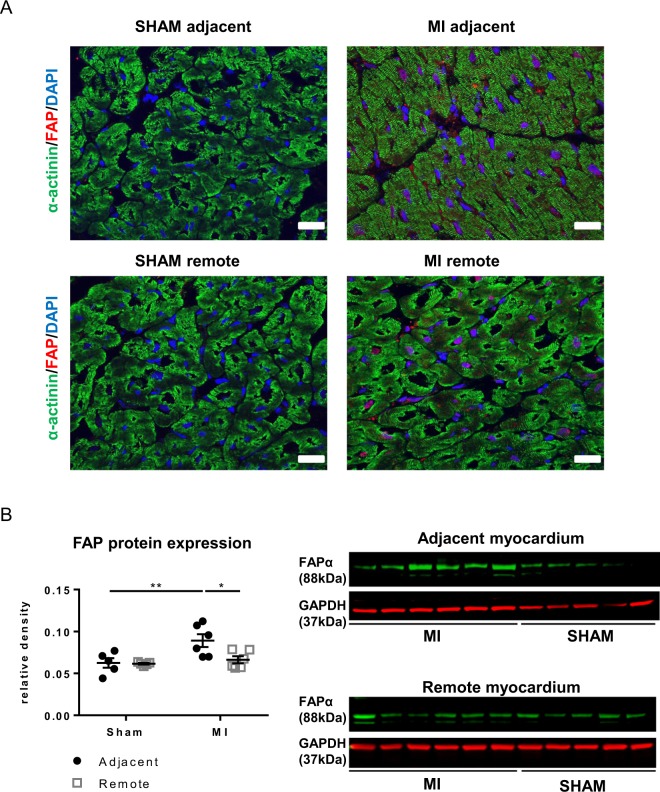
Myofibroblasts *in situ* in the adjacent myocardium. (**A**) Immunofluorescence staining of Fibroblast Activation Protein α, FAP-α, (red), α-actinin (green) and DAPI (blue) in SHAM and MI tissue sections. Scale bars represent 30 µm. (**B**) Western blotting of FAP-α (88-kDa) in tissue from the adjacent and remote myocardium of SHAM and MI. (*p < 0.05: **p < 0.01). (2-way ANOVA with Bonferroni post hoc test).

Fibroblast activation protein α (FAP-α), which is involved in extracellular matrix remodelling and cell migration^34^ has gained much interest as a specific marker of activated mature fibroblasts^35^. FAP can be identified *in situ* in sections of MI_adjacent_ and to a lesser extent in MI_remote_ (Fig. 3A); protein expression is significantly higher in the MI_adjacent_ but not in MI_remote_ compared to matched regions in SHAM (Fig. 3B).

### Stiffness of the adjacent and remote myocardium

Stiffening of the myocardium is related to an increased concentration of collagen type I^36^. Since collagen type I distribution is highly increased in the scar and moderately increased in MI_adjacent_, we analyzed regional passive mechanical properties of the LV myocardium. Supplemental Fig. 2A shows an example of tissue connected to the Biotester for biaxial stretching. Subjection of the tissues to a maximal load of 20 kPa induces a similar increase in compliance in the MI_adjacent_ and MI_remote_ compared to the matched regions in SHAM; as anticipated, scar tissue has a significant reduction in compliance (Supplemental Fig. 2B).

### Properties of fibroblasts isolated from different regions

**Figure 4:**
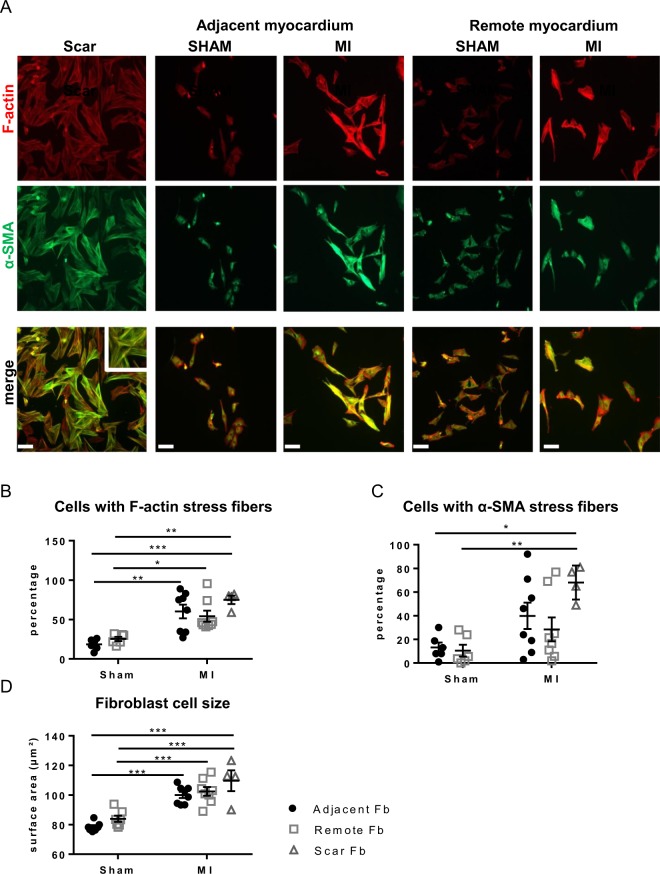
Differentiation of fibroblasts derived from scar, adjacent and remote myocardium. (**A**) Fluorescent images of fibroblastic cells stained for α-SMA (green) and F-actin (red) and the corresponding overlay after 4 days in culture. (**B**) Quantification of Fb positive for immuno-stained F-actin as percentage of all cells. (**C**) Quantification of α-SMA-positive Fb as percentage of all cells. (**D**) Cell size of Fb from SHAM and MI adjacent and remote myocardium. (*p < 0.05: **p < 0.01: *** < 0.001). (1-way ANOVA with Bonferroni post hoc test).

Fibroblast phenotypes isolated and cultured for 4 days (no passaging) from three regions of MI were compared to those from matched regions in SHAM (Fig. 4A). Fb from the scar are mainly MyoFb with F-actin stress fibers (75 ± 5% compared to 19 ± 3% in SHAM; Fig. 4B) decorated with α-SMA (68 ± 7% *vs*. 13 ± 4% in SHAM; Fig. 4C). A significant fraction of the Fb population of MI_adjacent_ (60 ± 9% *vs*. 19 ± 3% in SHAM) and MI_remote_ (54 ± 7% *vs*. 25 ± 3% in SHAM) likewise has F-actin stress fibers (Fig. 4B). However, there is no consistent presence of α-SMA with variability in the different samples of MI_adjacent_ and MI_remote_ (Fig. 4C). These MyoFb without α-SMA incorporation present thus as proto-MyoFb^7^. Both Fb from MI_adjacent_ and MI_remote_ exhibit a significant increase in cell size compared to SHAM (Fig. 4D). Cell cultures did not reach full confluence at the time of fixation. Counting of cell numbers at day 4 shows variability of cultures but on average there are no marked differences in cell density between the different groups (Supplemental Fig. 3) indicating cell density or contact inhibition are unlikely to influence potential differences in the fibroblast phenotype.

### Differentiated fibroblasts maintain their proliferative state but with variable contractile potential

**Figure 5:**
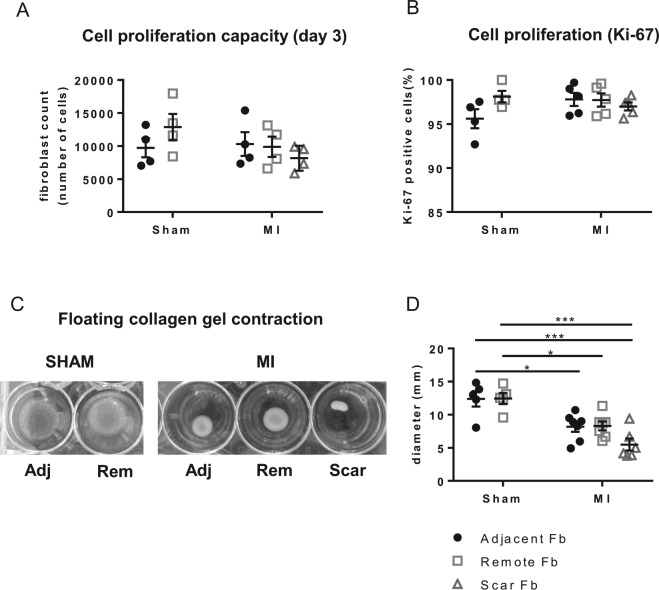
Proliferative and contractile properties of fibroblasts from the adjacent and remote myocardium. (**A**) Proliferation capacity of Fb, expressed as cell number after 3 days in culture with 5000 cells seeded at d0. (**B**) Percentage of Fb positive for Ki-67 marker. (**C**) Representative images of 3-DCM contraction by fibroblastic cells. (**D**) Analysis of the collagen gel diameter after 3 days in culture. (*p < 0.05: **p < 0.01: *** < 0.001). (1-way ANOVA with Bonferroni post hoc test).

Fb differentiation is associated with maturation of the stress fiber network and the progression into a senescent state^12, 37^. We have previously classified Fb differentiation based on the presence of a stress fiber network and their proliferative capacity i.e. undifferentiated proliferative Fb without stress fibers, differentiated proliferative myofibroblasts (p-MyoFb) and terminal differentiated non-proliferative myofibroblasts (non-p-MyoFb) with a well-developed stress fiber network^12^. These three phenotypes display distinct structural and functional characteristics here used as reference for the identification of the Fb phenotypes. MyoFb isolated from the scar continue to proliferate (Fig. 5A) after 3 days in culture, confirmed by the high number of cells positive for the proliferation marker Ki-67 (Fig. 5B, Supplemental Fig. 4). These MyoFb are therefore defined as proliferative MyoFb (p-MyoFb). Differentiated Fb from the MI_adjacent_ and MI_remote_ also maintain proliferation capacity (Fig. 5A) and Ki67 positivity (Fig. 5B, Supplemental Fig. 4) after 3 days in culture. These cell populations contain fractions of proto-MyoFb and p-MyoFb.

We further evaluated contraction of 3-dimensional collagen matrices (3-DCMs) by fibroblastic cells (Fig. 5C,D) which is directly proportional to the degree of Fb differentiation^38^. Scar derived p-MyoFb markedly reduce gel diameter by 56% compared to Fb from the matched SHAM region. 3-DCMs containing Fb from MI_adjacent_ achieve a 34% reduction in gel diameter compared to 3-DCM of SHAM. 3-DCMs embedded with Fb derived from MI_remote_ induce a reduction of the gel diameter by 33% compared to 3-DCMs of the matched SHAM region.

### Transcriptome analysis of the fibroblast phenotypes

We performed RNA sequencing on cultured Fb from the scar, from MI_adjacent_, from MI_remote_ and from the anterior region in SHAM (matching with MI_adjacent_), to identify region-specific transcriptional regulation. Comparison between all regions revealed a differential expression of a total of 1096 genes. We classified these genes into nine clusters (Supplemental Fig. 5A, Supplemental Table 1). When compared to SHAM_adjacent_, the genes related to Fb differentiation and matrix remodeling such as periostin (POSTN), TGF-β1, MMP-1 and TIMP-1 were significantly expressed in Fb from all regions of MI. Further analysis shows limited differences between MI_adjacent_ and MI_remote_ (Supplemental Fig. 5B) but a significant number of differentially expressed genes between Fb of scar and MI_adjacent_ (n = 43) and an even greater number between Fb of scar and MI_remote_ (n = 51) (Supplemental Fig. 5C,D). Fb from scar show an elevated expression of genes involved in ECM remodeling, such as periostin, matrix metalloproteinase enzymes (MMP-15, MMP-17), when compared to Fb of MI_adjacent_ or MI_remote_ (Supplemental Fig. 5C,D). Inflammatory cytokines such as interleukin-33 (IL-33), chemokine ligand 4 (CCl-4) are also found to be upregulated in scar Fb. A limited but significant number of genes such as for periostin, fibrillin and HtrA serine peptidase 3 are differentially regulated between Fb of MI_adjacent_ and MI_remote_. Taken together, the cultured Fb phenotypes indicate that Fb from MI_adjacent_ and MI_remote_ are activated towards a MyoFb phenotype with limited differences between the regions in these in vitro conditions. We thus further examined in tissue samples local signals for this activation and the differences between MI_adjacent_ and MI_remote_ *in situ* in the heart.

### TGF-β1 activation in the adjacent and remote myocardium *in situ*

**Figure 6:**
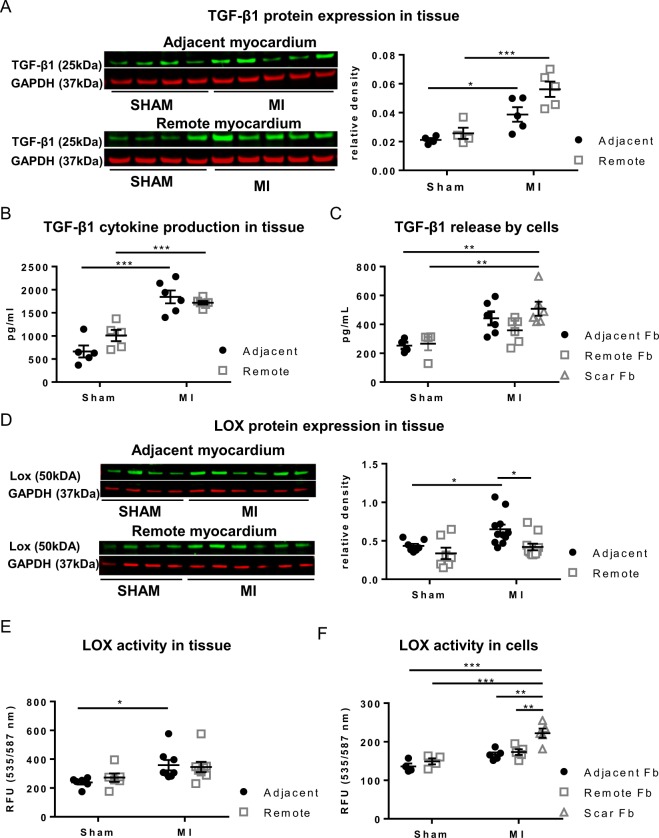
TGF-β1 and LOX expression during post-MI remodeling. (**A**) Protein expression by Western blotting of TGF-β1. (**B**) TGF-β1 concentration measured in cytokine array. (**C**) TGF-β1 secretion by cultured Fb cells derived from SHAM and MI. (**D**) LOX expression in tissue from the adjacent and remote myocardium of SHAM and MI. (**E**) LOX activity in tissue, (**F**) Lox activity in cultured Fb cells derived from SHAM and MI. *p < 0.05, **p < 0.01. (*p < 0.05: **p < 0.01: *** < 0.001). (2-way ANOVA with Bonferroni post hoc test for 6A, B, D, E;1-way ANOVA with Bonferroni post hoc test for 6 C, F)

We determined the protein expression of TGF-β1, since it plays an important role in the pathogenesis of cardiac remodeling and fibrosis^39^. Protein expression of TGF-β1 is significantly increased in tissue lysates of MI_adjacent_ (1.8 fold increase) and MI_remote_ (2.1 fold increase) compared to matched regions in SHAM (Fig. 6A). Western blot results are supported by the cytokine measurement in which similar increases in TGF-β1 concentration are observed in MI_adjacent_ and MI_remote_ compared to SHAM (Fig. 6B). We further quantified the amount of TGF- β1 released by cultured Fb. This data show a marked secretion of TGF-β1 by the scar derived Fb compared to Fb from the matched SHAM regions (Fig. 6C). A tendency for increased TGF-β1 secretion is noted by Fb from the MI_adjacent_.

### Regional expression and activation of lysyl oxidase

**Figure 7:**
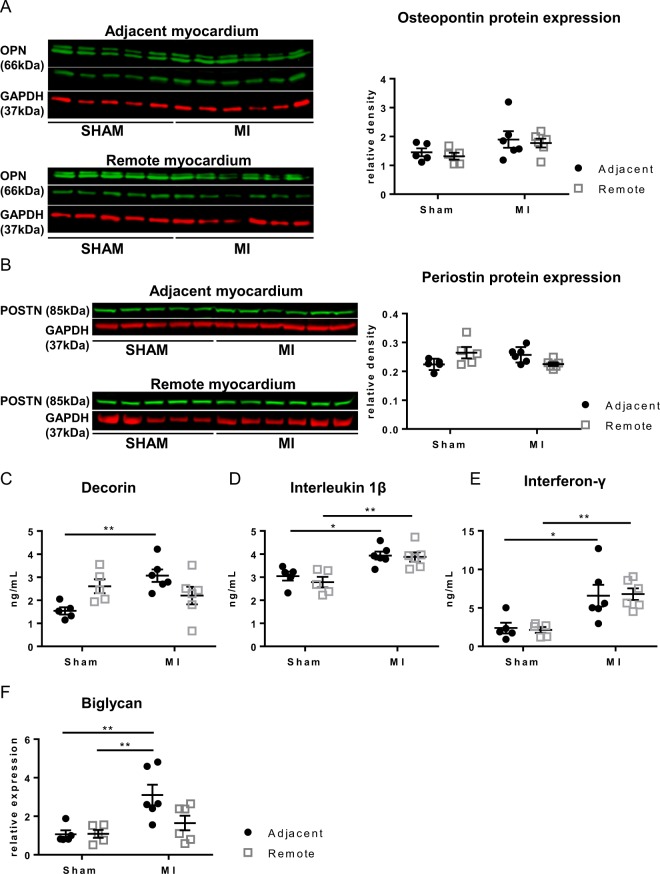
Cytokines in the adjacent and remote myocardium of SHAM and MI. (**A**,**B**) Cytokines assessed with Western blotting for osteopontin and periostin in tissue from the adjacent and remote myocardium. (**C**–**E**) Assessment in cytokine array of secretion of decorin (**C**), interleukin 1β (**D**), interferon-γ (**E**). (**F**) mRNA expression of biglycan (*p < 0.05: **p < 0.01). (2-way ANOVA with Bonferroni post hoc test).

LOX plays a pivotal role in collagen cross-linking^15^. We determined LOX protein expression and activity in tissue lysates and compared those to matched regions in SHAM. Increased expression of the uncleaved intracellular LOX pro-enzyme is observed in the MI_adjacent_ (1.4 fold increase) but not in MI_remote_, when compared to matched regions in SHAM (Fig. 6D). Concomitantly, a significant upregulation of LOX activity, which measures extra-cellular LOX is only detected within MI _adjacent_ (1.5 fold increase; Fig. 6E). Whether the Fb are a source of LOX is hard to measure in the tissue samples. However, LOX activity in cultured Fb from matched samples is increased only in p-MyoFb from the scar, whereas LOX activity is similar between cells from MI_adjacent_ and MI_remote_ and Fb from matched regions in SHAM (Fig. 6F). Activation of LOX requires a conformational change triggered by either osteopontin (OPN)^40^ and/or periostin^41^. RNA sequencing reveals a significant upregulation of periostin mRNA expression in p-MyoFb from the scar but not in MI_adjacent_ or MI_remote_. We analyzed the protein expression of periostin and OPN in tissue lysates but no differences were observed in MI_adjacent_ or in MI_remote_ (Fig. 7A,B).

### Differential production of cytokines in the adjacent and remote myocardium

ECM remodeling after MI is associated with differential expression of cytokines and growth factors. We performed a porcine Quantibody protein array on tissue lysates in which different cytokines were quantified simultaneously (Fig. 7C–E). Elevated levels of inflammatory cytokines such as interleukin 1β (IL1β) and interferon γ (IFNγ) are found in the MI_adjacent_ and MI_remote_ regions.Decorin, a small leucine-rich proteoglycan, which plays an important role in collagen fibrillogenesis, is significantly upregulated in MI_adjacent_. In addition, we evaluated mRNA expression of biglycan, another small leucine-riche proteoglycan similar to decorin, showing increased expression in MI_adjacent_ (Fig. 7F).

## Discussion

In the pig with MI and replacement fibrosis in the scar, interstitial and perivascular fibrosis are also present, in the MI_adjacent_ but not in the MI_remote_. However, Fb isolated from both regions show similar differentiation towards the MyoFb phenotype. MyoFb from the scar have a more pronounced differentiation and contractile phenotype. In MI_adjacent_, collagen cross-linking can be seen and may be driven by local LOX.

### Fibroblast activation throughout the LV

In agreement with other studies^20^, we find that Fb derived from scar tissue are MyoFb with a well-developed F-actin and α-SMA stress fiber network, and with the capacity to contract the ECM. Although it was reported in the mouse that MI induces senescence in Fb in the infarct and border zone^42^, here we found that MyoFb from the scar maintained their proliferation capacity. This is likely due to the ongoing proliferative phase of the scar tissue, which requires active synthesis of ECM proteins^43^. Scar and replacement fibrosis are thus the result of a specific differentiated MyoFb population.

FAP-α staining *in situ* suggests that MyoFb differentiation is present in MI_adjacent_ but is less pronounced in the MI_remote_. Yet, Fb isolated from both regions and cultured for 4 days were not substantially different: both had characteristics of proliferative MyoFb with the potential to contract collagen matrices but to a lesser extent than scar MyoFb. These data therefore suggest that even if there is little evidence for fibrosis or strong presence of MyoFb in situ in the MI_remote_, Fb in these regions are primed and activated. Such activation may result from the mechanical load that is increased in both the MI_adjacent_ and MI_remote_ compared to SHAM^32^. Mechano-signal transduction is propagated by integrins located at focal adhesions of Fb, where they aid in the release of latent TGF-β1 from the matrix, which in turn promotes Fb differentiation^7^. In support of this, blocking of integrin αvβ3 and αvβ5 has recently been shown to suppress Fb differentiation^13^. Analysis of TGF-β1 protein expression at the cellular and tissue level indeed confirmed a marked increase in both non-infarcted regions, which suggests that global Fb differentiation is driven by TGF-β1 associated signaling pathways. The role of downstream signaling from TGF-beta in cardiac Fb differentiation has previously been shown in rat cardiac Fb^12^ where inhibition of TGF-β1R kinase can convert the MyoFb phenotype towards that of a non-differentiated Fb and this is also the case in the pig heart (ongoing work).

Of note, transcriptome analysis could detect differences in gene expression between Fb from MI_adjacent_ and MI_remote_ but this did not reflect in a substantial difference in phenotype.

### Region-specific fibrosis

In the scar, replacement fibrosis is associated with high levels of collagen type I and III fibers deposited by p-MyoFb. The scar derived p-MyoFb secrete high levels of TGF-β1 resulting in a self-perpetuating process of Fb differentiation and extracellular matrix remodeling. Despite evidence for global Fb activation, significant interstitial and perivascular fibrosis is seen only in MI_adjacent_ and not in MI_remote_. This suggests that a more mature MyoFb phenotype is present in MI_adjacent_ and that there are local cues that are lost when Fb are isolated and cultured. Of note, there are data which show that interstitial fibrosis is not directly linked to an increased number of α-SMA positive MyoFb, as reported in a model of pressure-overload^28^. This is further substantiated by a recent study in which FAP-α positive Fb secrete collagen independent of α-SMA expression^35^. Expression of FAP-α has been designated as a more reliable marker for mature MyoFb differentiation. This is in line with the present data for in situ staining and Western blot showing increased FAP-α expression in MI_adjacent_ but not in MI_remote_ regions, concomitant with fibrosis in MI_adjacent_ only. When compared to the rat model of acute MI from Tillmanns *et al*., FAPα expression levels are rather moderate^35^. A possible explanation is the more gradual development and smaller infarct size in our model as well as a reduction in the pro-inflammatory state at the time-point of measurement. Notably, levels of TGF- β1, IL-1β and IFNγ are all elevated in both the MI_adjacent_ and MI_remote_ regions indicating a pro-inflammatory environment for MyoFb activation^44, 45^ suggesting that the activation of collagen secretion in the MI_adjacent_ requires additional signals. The proximity of the scar could lead to diffusion of signals and/or more pronounced local activation that promotes this local fibrosis in MI_adjacent_. This local signaling superimposes on a MyoFb phenotype that when studied *ex vivo* is not very different between MI_adjacent_ and MI_remote_.

It should be emphasized that the MI_adjacent_ is not inter-digitating with scar tissue and that the observed interstitial and perivascular fibrosis is not replacement fibrosis. However, in the pig with severe left anterior descending (LAD) stenosis, this adjacent region has reduced perfusion reserve and Fb from the MI_adjacent_ may experience oxidative stress, which can lead to perivascular fibrosis^46^. Indeed, when Fb are exposed to reactive oxygen species in culture, they increase the production of ECM proteins and the secretion of collagen^47^.

Although wall stress is increased throughout the LV compared to SHAM, close to the infarct dynamic effects because of tethering to the non-contractile scar may increase the mechanical load on the MI_adjacent_. In a recent study using cellular hypertrophy and T-tubule remodeling as a read-out, Frisk and colleagues argued for such increased local stress, though differences were small^48^.

Local molecular activation of the copper dependent amine oxidase LOX, which is also secreted by MyoFb^17, 49^ promotes collagen cross-linking, ultimately leading to a stiffer scar, which is resistant to MMPs^50^. p-MyoFb from scar tissue demonstrate a high capacity to activate LOX and thus to promote cross-linked collagen. The moderate increase in interstitial fibrosis and highly cross-linked collagen type I in the MI_adjacent_ could be related to the region-specific increase in LOX concentration and enzymatic activity within the tissue. LOX is activated via increased synthesis of matricellular proteins such as osteopontin and periostin^40, 41^. Both are not changed in the MI_adjacent_ but the proteins could be below detection levels. Of note, in cultured Fb from the scar and MI_adjacent_ RNA sequencing indicates an increase in periostin expression. Furthermore, biglycan and decorin levels are higher in the MI_adjacent_ and could support the assembly of the ECM and the stabilization of collagen fibrillogenesis. The role of decorin has previously been studied in a chronic model of myocardial infarction showing increased concentrations within the scar, border zone and the remaining viable myocardium after 8 weeks post-MI^51^. Similarly, biglycan is associated with elevated cardiac fibrosis and displays similar functional characteristics as decorin^52, 53^. Beyond local production by the MyoFb as a result of oxidative stress and mechanical load, it is also possible that the source of these proteoglycans is the more mature MyoFb population in the scar with diffusion into the adjacent myocardium.

It is conceivable that some of the signals are transient and peak before the 6 weeks. To detect early activation of collagen cross-linking, we also evaluated osteopontin protein expression at 2 weeks of MI but found no evidence for increased levels (data not shown).

### Limitations

Our study provides a cross-sectional view in a large animal model; while this allows a regional analysis combining samples from the same animal for different parameters, interventions targeting mechanisms are difficult. Intrinsically, the study of Fb without affecting the *in vivo* phenotype is challenging. Access *in situ* is difficult and without culturing the number of cells that is recovered is limited.

We focused on the Fb phenotype and collagen deposition, though it is possible that differences in matrix remodeling by MMPs and tissue inhibitors of metalloproteinase (TIMPs) is different in the regions. Several questions related to the regional molecular mechanisms remain to be investigated and may require more in depth cell-specific signatures and more complex studies in multicellular preparations.

### Conclusions and perspectives

After MI, Fb are activated throughout the LV, presumably through TGF-β1 in response to increased wall stress and/or inflammation. Additional regional specific signaling leads to interstitial fibrosis via collagen cross-linking in MI_adjacent_. Local LOX activity could be stimulated by higher mechanical load imposed by tethering to the infarct or signals could diffuse from the scar. Identifying specific signaling cues to maintain the mature state of MyoFb phenotype in the scar tissue may open new perspectives in targeting the MyoFb reversibility in interstitial fibrosis without damaging existing scar tissue.

## Material and Methods

### Pig model of ischemic cardiomyopathy

All experiments were performed in accordance with the European Directive 2010/63/EU. The animal experimental protocol was approved by the Ethical Committee for Animal Experiments of the KU Leuven, Belgium, with permit numbers P10139 and P14176.

Domestic pigs (weight between 20–25 kg, n = 11) were implanted with a copper-coated stent into the LAD coronary artery, which leads to a severe coronary stenosis and MI as described before^32^. The same procedure was repeated without stent implantation to generate SHAM animals (n = 7). 6 weeks after stent implantation, global and regional left ventricular functions were assessed by 3T MRI (TRIO-tim, Siemens, Erlangen, Germany). MRI analysis showed a decrease in ejection fraction (EF), end-diastolic (EDV) and end-systolic volume (ESV) in MI animals (Supplemental Fig. 6A). The presence and extent of MI was determined from delayed enhancement images. The global end-diastolic and end-systolic wall stress were significantly higher in these MI animals (Supplemental Fig. 6B). Previously, we documented that adenosine infusion resulted in a reduction in perfusion reserve in the infarct and adjacent myocardium of MI animals, whereas baseline perfusion was reduced only in the infarct region; dobutamine stress challenge showed a reduction in wall thickening in the adjacent and to a lesser extent in the remote myocardium compared to SHAM animals^32^.

### Tissue samples, fibroblast isolation and culture

6 weeks after stent implantation, animals were sacrificed and heart tissue was obtained for tissue sampling and cell isolation. Animals were pre-anesthetized with tiletamine/zolazepam (8 mg/kg IM, Virbac, Leuven, Belgium) and xylazine (2.5 mg/kg IM, VMD, Arendonk, Belgium) and sacrificed with an overdose of pentobarbital (100 mg/kg IV, Ceva, Brussels, Belgium). Tissues samples were taken from 3 LV regions (Supplemental Fig. 6C) i.e. (i) the scar tissue, (ii) the anterior myocardium perfused by the LAD coronary artery adjacent to the infarction (MI_adjacent_) and (iii) the posterior myocardium perfused by the left circumflex coronary artery (LCX) remote from the infarction (MI_remote_). In SHAM animals, samples were taken from matched regions. A number of samples were fixed in 4% paraformaldehyde (PFA) and stored for later processing for histological analysis. Tissue samples for Fb isolation were processed immediately. A biopsy of approximately 750–1000 mg was cut into small pieces using a surgical blade followed by washing in Tyrode’s solution (in mmol/L: NaCl 137, KCl 5.4, MgCl_2_ 0.5, CaCl_2_ 1.8, Na-HEPES 11.8, and glucose 10; pH 7.4) for 10 minutes. Tissue digestion was performed in 20 ml of nominally Ca^2+^-free Tyrode’s solution containing 28 mg (0.229 U/mg) of collagenase-A (Roche, 10103586001) and 7 mg protease XIV (Sigma P5147), during 20 minutes followed by a second digestion step in 30 ml of nominally Ca^2+^-free Tyrode’s solution containing 28 mg (0.229 U/mg) of collagenase-A without protease for 30 minutes. The digested tissue was minced and the obtained cell suspension was filtered to remove cell debris (200 µm mesh size). The cell suspension containing Fb and cardiac myocytes was centrifuged and the cell pellet was plated onto a 6-well plate (Corning). Since cardiac myocytes couldn’t attach to the culture plate, these cells were removed by a washing step. Cells were cultured with DMEM (Thermofisher, DMEM 22320-030) supplemented with 10% fetal bovine serum (Gibco, Invitrogen), and 1% of penicillin/streptomycin (Invitrogen) solution for 4 days.

### Immunofluorescence staining of cells and tissue for evaluation of Fb differentiation

At day 4 of culture, Fb were fixed for 20 minutes in 1% PFA diluted in Phosphate-buffered saline (PBS). Cells were double stained for F-actin stress fibers using Rhodamin-Phalloidin (A12380, Invitrogen, Molecular Probes) and for α-SMA stress fibers (A2547, Sigma). Fluorescent images were collected with an Axiocam HrC camera mounted on a Zeiss Axioplan microscope and data was recorded using the Axiovision software program. Subsequently, cell size was measured in ten randomly chosen images for each sample. Fb differentiation was evaluated by counting the number of cells positive for either F-actin or α-SMA stress fibers. In order to evaluate the proliferation capacity, 24-well culture plates (Corning) were seeded with 5000 Fb cells per well. After 3 days in culture, number of cells was counted in a Bürker counting chamber. Also, cell proliferation was assessed by labeling Fb cells after 4 days in culture with Ki-67 antibody (ab15580, Abcam) and percentage of Ki-67 positive cells was calculated by normalizing it to total the number of DAPI positive nucleus.

To examine Fb differentiation *in situ*, cryo-sections of tissue samples taken at the time of sacrifice were fixed and permeabilized and then non-specific sites blocked with goat serum. Subsequently, the sections were incubated overnight with primary antibodies against FAP-α (orb97039, Biorbyt) and alpha-actinin (A5044, Sigma). The next day, sections were washed with PBS and incubated with secondary antibodies (A32723, Alexa 488 conjugated goat anti-mouse, A10042, Alexa 568 conjugated donkey anti-rabbit, Invitrogen). The tissue sections were finally embedded with Prolong Gold Antifade reagent containing DAPI (ThermoFisher Scientific). Morphometry was performed using ImageJ analysis software (National Institutes of Health, http://rsb.info.nih.gov/ij/).

### Myofibroblast contractile properties

To evaluate the functional capacity to contract 3-DCM, 50.000 Fb at day 4 of culture from SHAM or MI were mixed in rat tail collagen type I (1.5 mg/ml) (Corning) and Dulbecco’s Modified Eagle Medium (Sigma, cat. no. D2429). The collagen mixture was transferred to 24-well plates (Corning) coated with 1% BSA. After polymerization for 1 hour at 37 °C, DMEM without FBS was added to the culture well releasing the collagen gel from the culture plate. Floating collagen gels were maintained in culture for 3 days. Gels were scanned at day 3 and contraction was evaluated using ImageJ by measuring the diameter of the gels.

### Illumina high-throughput sequencing and gene expression analysis

Total RNA of Fb cell samples was isolated and purified using the RNeasy mini kit according to the manufacturer’s instructions (Qiagen). RNA concentration and integrity was determined using the Nanodrop ND-1000 (Nanodrop technologies) and the Bioanalyser 2100 (Agilent). Stranded mRNA libraries were prepared according to manufacturer’s instructions (TrueSeq, Illumina). The prepared libraries were sequenced on a HiSeq. 2500 platform. The 50 BP tags were mapped to the reference pig genome Sscrofa 10.2 using the RNA-Seq by Expectation Maximization (RSEM)^54^ pipeline with Spliced Transcripts Alignment to a Reference (STAR)^55^ as an aligner. The read counts and FPKM values were given as input to the Trinity package^56^ for identification of differentially expressed genes. The molecular signature database^57, 58^ was used for the identification of significantly enriched gene ontology terms related to biological processes. The differentially expressed genes were identified by comparing the expression in different tissues to SHAM. All RNA-seq data will be publicly available for download from the Gene Expression Omnibus on publication (GSE98504).

### Quantification of fibrosis *in situ*

Samples fixed in 4% paraformaldehyde in PBS were processed and embedded in paraffin. Consequently, 8 µm thick sections were deparaffinized, rehydrated and stained for collagen using the PicroSirius red staining kit (PolySciences). After staining, sections were mounted in DPX. Images were acquired using the Zeiss Axioplan microscope with the Axiocam HrC camera. Polarization microscopy was performed on the Sirius red stained sections to visualize collagen type I and III based on the birefringence properties of collagen. The total amount of fibrosis and collagen type I and III were quantified using the Axiovision software analysis program.

### Electron microscopy

LV samples were fixed in 3% glutaraldehyde (Sigma) with 0.1 M KH_2_PO_4_, pH 7.4 and post-fixed for 1 hour with 2% OsO_4_ in 0.1 M sodium cacodylate. Samples were dehydrated in graded series of ethanol and embedded in Epoxy resin (Science Services, München, Germany). Ultra-thin sections of epoxy resin blocks (2 µm) were counterstained with uranyl acetate and lead citrate prior to examination in a JEOL 2100 electron microscope (JEOL, Zaventem, Belgium).

### Immunoblotting for protein expression

After sacrifice, LV tissue samples were collected and immediately cryo-preserved in liquid nitrogen and stored at −80 °C. Total protein lysates were prepared as described before^59^. Equal amounts of proteins were separated on a 4–12% Bis-Tri gradient gel (Invitrogen) and transferred onto polyvinylidene difluoride membrane (Millipore). The membrane was blocked with Odyssey blocking buffer (Li-cor) and probed for lysyl oxidase (LOX), (sc-373995 Santa Cruz), Periostin (ab79946, Abcam), TGF-β1 (ab92486, Abcam), Osteopontin (ab8448, Abcam), Fibroblast activation protein-α (orb97039, Biorbyt) and GAPDH (sc-25778, Santa Cruz). Immunofluorescence detection was performed with matching secondary antibodies (Li-cor 800CW or Alexa Fluor 680, ThermoFisher Scientific) using an Infrared imaging system (Li-cor, CLx).

### Quantitative RT-PCR

RNA was isolated from the tissue, by lysing the small tissue pieces using lysing matrix tubes in (MP Biomedicals) in TRIzol (Ambion Thermo Scientific), followed by phenol-chloroform total RNA isolation (according manufacturer’s instructions). RNA was quantified using the Nanodrop™ 2000c spectrophotometer (Thermo Scientific) and 500 ng of RNA was used for cDNA synthesis with Superscript II (Thermo Scientific). The final cDNA reaction was diluted 1:20 in molecular biology grade water (Thermo Scientific). qRT-PCR was performed on a 7500 Fast Real-Time PCR machine using the SYBRGreen master mix (Applied Biosystems, Belgium). The relative gene expression was calculated by comparing cycle times for target PCR using the following equation: relative gene expression = 2 – (ΔCtsample − ΔCtcontrol).Values are normalized to housekeeping gene RPL-32 expression levels. Primer sequences for collagen type I, collagen type III, biglycan and RPL-32 can be found in Supplemental Table 2.

### Lysyl oxidase activity

LOX activity was fluorometrically measured in total protein lysates from tissue and cell culture samples using the LOX activity assay (AAT Bioquest) according to the manufacturer’s instructions. Measurement was performed using the FlexStation 2 microplate reader (Molecular Devices).

### Cytokine quantification

Cytokine concentration was measured in tissue lysates using the Quantibody Porcine Cytokine Array 1 and 3 (RayBiotech) according to the manufacturer’s instructions. Fluorescence detection of cytokines was performed on a GenePix 4000B Microarray scanner. Only cytokines with robust signals above the detection limit of the assay were included in the analysis. ELISA was performed to quantify the secretion of TGF-β1 by fibroblasts in conditioned media after 4 days in culture according to manufacturer’s instructions (R&D systems MB100B). The concentration was then normalized to the number of cells in culture.

### Statistics

Statistical analysis was performed using one-way ANOVA or two-way ANOVA with Bonferroni correction. For analysis of images and molecular readouts, the operator was blinded to the data type by coding of samples. All data are expressed as mean ± SEM.

## Electronic supplementary material

Supplementary Information

## References

[1] Pinto AR (2016). Revisiting Cardiac Cellular Composition. Circ. Res..

[2] Krenning G, Zeisberg EM, Kalluri R (2010). The origin of fibroblasts and mechanism of cardiac fibrosis. J. Cell. Physiol..

[3] Ieda M (2009). Cardiac fibroblasts regulate myocardial proliferation through beta1 integrin signaling. Dev. Cell.

[4] Takeda N (2010). Cardiac fibroblasts are essential for the adaptive response of the murine heart to pressure overload. J. Clin. Invest..

[5] Ali SR (2014). Developmental heterogeneity of cardiac fibroblasts does not predict pathological proliferation and activation. Circ. Res..

[6] Burstein B, Libby E, Calderone A, Nattel S (2008). Differential behaviors of atrial versus ventricular fibroblasts: a potential role for platelet-derived growth factor in atrial-ventricular remodeling differences. Circulation.

[7] Tomasek JJ, Gabbiani G, Hinz B, Chaponnier C, Brown RA (2002). Myofibroblasts and mechano-regulation of connective tissue remodelling. Nat. Rev. Mol. Cell Biol..

[8] MacKenna D, Summerour SR, Villarreal FJ (2000). Role of mechanical factors in modulating cardiac fibroblast function and extracellular matrix synthesis. Cardiovasc. Res..

[9] Souders CA, Bowers SLK, Baudino TA (2009). Cardiac fibroblast: the renaissance cell. Circ. Res..

[10] Schroer AK, Merryman WD (2015). Mechanobiology of myofibroblast adhesion in fibrotic cardiac disease. J. Cell Sci..

[11] Bai J (2013). Metformin inhibits angiotensin II-induced differentiation of cardiac fibroblasts into myofibroblasts. PloS One.

[12] Driesen RB (2014). Reversible and irreversible differentiation of cardiac fibroblasts. Cardiovasc. Res..

[13] Sarrazy V (2014). Integrins αvβ5 and αvβ3 promote latent TGF-β1 activation by human cardiac fibroblast contraction. Cardiovasc. Res..

[14] Agarwal SK (2014). Integrins and cadherins as therapeutic targets in fibrosis. Front. Pharmacol..

[15] López B (2010). Role of lysyl oxidase in myocardial fibrosis: from basic science to clinical aspects. Am. J. Physiol. Heart Circ. Physiol..

[16] Adam O (2011). Increased lysyl oxidase expression and collagen cross-linking during atrial fibrillation. J. Mol. Cell. Cardiol..

[17] Noppe G (2014). Reduced scar maturation and contractility lead to exaggerated left ventricular dilation after myocardial infarction in mice lacking AMPKα1. J. Mol. Cell. Cardiol..

[18] Van Aelst LNL (2015). Osteoglycin prevents cardiac dilatation and dysfunction after myocardial infarction through infarct collagen strengthening. Circ. Res..

[19] Sun Y, Kiani MF, Postlethwaite AE, Weber KT (2002). Infarct scar as living tissue. Basic Res. Cardiol..

[20] Willems IE, Havenith MG, De Mey JG, Daemen MJ (1994). The alpha-smooth muscle actin-positive cells in healing human myocardial scars. Am. J. Pathol..

[21] Rog-Zielinska EA, Norris RA, Kohl P, Markwald R (2016). The Living Scar–Cardiac Fibroblasts and the Injured Heart. Trends Mol. Med..

[22] Stefanon I (2013). Left and right ventricle late remodeling following myocardial infarction in rats. PloS One.

[23] Voorhees AP (2015). Building a better infarct: Modulation of collagen cross-linking to increase infarct stiffness and reduce left ventricular dilation post-myocardial infarction. J. Mol. Cell. Cardiol..

[24] Olivetti G, Capasso JM, Sonnenblick EH, Anversa P (1990). Side-to-side slippage of myocytes participates in ventricular wall remodeling acutely after myocardial infarction in rats. Circ. Res..

[25] Taniyama Y (2016). Selective Blockade of Periostin Exon 17 Preserves Cardiac Performance in Acute Myocardial Infarction. Hypertens. Dallas Tex 1979.

[26] Moore-Morris T, Cattaneo P, Puceat M, Evans SM (2016). Origins of cardiac fibroblasts. J. Mol. Cell. Cardiol..

[27] Kanisicak O (2016). Genetic lineage tracing defines myofibroblast origin and function in the injured heart. Nat. Commun..

[28] Moore-Morris T (2014). Resident fibroblast lineages mediate pressure overload-induced cardiac fibrosis. J. Clin. Invest..

[29] Zeisberg EM (2007). Endothelial-to-mesenchymal transition contributes to cardiac fibrosis. Nat. Med..

[30] Widyantoro B (2010). Endothelial cell-derived endothelin-1 promotes cardiac fibrosis in diabetic hearts through stimulation of endothelial-to-mesenchymal transition. Circulation.

[31] Heinzel FR (2008). Remodeling of T-tubules and reduced synchrony of Ca2+ release in myocytes from chronically ischemic myocardium. Circ. Res..

[32] Galan DT (2016). Reduced mitochondrial respiration in the ischemic as well as in the remote non-ischemic region in post-myocardial infarction remodeling. Am. J. Physiol. Heart Circ. Physiol..

[33] Dawson K, Wu C-T, Qi XY, Nattel S (2012). Congestive heart failure effects on atrial fibroblast phenotype: differences between freshly-isolated and cultured cells. PloS One.

[34] Kelly T, Huang Y, Simms AE, Mazur A (2012). Fibroblast activation protein-α: a key modulator of the microenvironment in multiple pathologies. Int. Rev. Cell Mol. Biol..

[35] Tillmanns J (2015). Fibroblast activation protein alpha expression identifies activated fibroblasts after myocardial infarction. J. Mol. Cell. Cardiol..

[36] Badenhorst D (2003). Cross-linking influences the impact of quantitative changes in myocardial collagen on cardiac stiffness and remodelling in hypertension in rats. Cardiovasc. Res..

[37] Goffin JM (2006). Focal adhesion size controls tension-dependent recruitment of alpha-smooth muscle actin to stress fibers. J. Cell Biol..

[38] Lijnen P, Petrov V, Fagard R (2001). *In vitro* assay of collagen gel contraction by cardiac fibroblasts in serum-free conditions. Methods Find. Exp. Clin. Pharmacol..

[39] Lijnen PJ, Petrov VV, Fagard RH (2000). Induction of cardiac fibrosis by transforming growth factor-beta(1). Mol. Genet. Metab..

[40] López B (2013). Osteopontin-mediated myocardial fibrosis in heart failure: a role for lysyl oxidase?. Cardiovasc. Res..

[41] Maruhashi T, Kii I, Saito M, Kudo A (2010). Interaction between periostin and BMP-1 promotes proteolytic activation of lysyl oxidase. J. Biol. Chem..

[42] Zhu F (2013). Senescent cardiac fibroblast is critical for cardiac fibrosis after myocardial infarction. PloS One.

[43] Chen W, Frangogiannis NG (2013). Fibroblasts in post-infarction inflammation and cardiac repair. Biochim. Biophys. Acta.

[44] Kawaguchi M (2011). Inflammasome activation of cardiac fibroblasts is essential for myocardial ischemia/reperfusion injury. Circulation.

[45] Siwik DA, Chang DL, Colucci WS (2000). Interleukin-1beta and tumor necrosis factor-alpha decrease collagen synthesis and increase matrix metalloproteinase activity in cardiac fibroblasts *in vitro*. Circ. Res..

[46] Zhao W, Zhao T, Chen Y, Ahokas RA, Sun Y (2008). Oxidative stress mediates cardiac fibrosis by enhancing transforming growth factor-beta1 in hypertensive rats. Mol. Cell. Biochem..

[47] Anupama V (2016). Molecular mechanisms in H2O2-induced increase in AT1 receptor gene expression in cardiac fibroblasts: A role for endogenously generated Angiotensin II. J. Mol. Cell. Cardiol..

[48] Frisk M (2016). Elevated ventricular wall stress disrupts cardiomyocyte t-tubule structure and calcium homeostasis. Cardiovasc. Res..

[49] Yang J (2016). Targeting LOXL2 for cardiac interstitial fibrosis and heart failure treatment. Nat. Commun..

[50] Sivakumar P, Gupta S, Sarkar S, Sen S (2008). Upregulation of lysyl oxidase and MMPs during cardiac remodeling in human dilated cardiomyopathy. Mol. Cell. Biochem..

[51] Hao J (1999). Elevation of expression of Smads 2, 3, and 4, decorin and TGF-beta in the chronic phase of myocardial infarct scar healing. J. Mol. Cell. Cardiol..

[52] Beetz N (2016). Ablation of biglycan attenuates cardiac hypertrophy and fibrosis after left ventricular pressure overload. J. Mol. Cell. Cardiol..

[53] Westermann D (2008). Biglycan is required for adaptive remodeling after myocardial infarction. Circulation.

[54] Li B, Dewey CN (2011). RSEM: accurate transcript quantification from RNA-Seq data with or without a reference genome. BMC Bioinformatics.

[55] Dobin A (2013). STAR: ultrafast universal RNA-seq aligner. Bioinforma. Oxf. Engl..

[56] Grabherr MG (2011). Full-length transcriptome assembly from RNA-Seq data without a reference genome. Nat. Biotechnol..

[57] Subramanian A (2005). Gene set enrichment analysis: a knowledge-based approach for interpreting genome-wide expression profiles. Proc. Natl. Acad. Sci. U. S. A..

[58] Mootha VK (2003). PGC-1alpha-responsive genes involved in oxidative phosphorylation are coordinately downregulated in human diabetes. Nat. Genet..

[59] Lenaerts I (2013). Role of nitric oxide and oxidative stress in a sheep model of persistent atrial fibrillation. Eur. Eur. Pacing Arrhythm. Card. Electrophysiol. J. Work. Groups Card. Pacing Arrhythm. Card. Cell. Electrophysiol. Eur. Soc. Cardiol..

