# Supplementary material for: Global fibroblast activation throughout the left ventricle but localized fibrosis after myocardial infarction
Source: Sci Rep. 2017 Sep 7;7:10801. doi: 10.1038/s41598-017-09790-1 (PMC5589875; doi:10.1038/s41598-017-09790-1)
Supplement: Supplementary file 1 — Supplementary Information [file 41598_2017_9790_MOESM1_ESM.pdf]

Online Supplemental Material and Methods to

Global fibroblast activation throughout the left ventricle but localized fibrosis after myocardial infarction

Chandan K. Nagaraju[1], Eef Dries[1], Natasa Popovic[2], Abhishek A. Singh[1], Peter Haemers[1], H. Llewelyn Roderick[1], Piet Claus[2], Karin R. Sipido[1,#,*], Ronald B. Driesen1[*]

*Equal contribution

[1] Division of Experimental Cardiology and [2] Cardiovascular Imaging and Dynamics, Department of Cardiovascular Sciences, KU Leuven, University of Leuven, Belgium

Author contributions: Study design KRS,RBD,PC,CKN, HLR; experimental data acquisition & analysis CKN, NP, AAS, ED, PH; primary manuscript drafting CKN, RBD, KRS, PC, HLR; final manuscript and approval - all.

Abbreviated title: Regional fibroblast differentiation after myocardial infarction

Key words: myocardial infarction, fibrosis, fibroblasts, hemodynamic load, ischemia, LOX

#

Address for correspondence:

Karin R. Sipido, MD, PhD

Department of Cardiovascular Sciences, Division of Experimental Cardiology

KU Leuven

Campus Gasthuisberg, Herestraat 49

B-3000 Leuven, Belgium

E-mail: karin.sipido@kuleuven.be

Extended methods

Mechanical testing of left ventricular tissue deformation

To evaluate the stiffness of the tissue, unfixed fresh samples were embedded in 6% agar (USB) diluted in 0% Calcium Normal Tyrode. Prior to embedding, the agar solution was heated to reach a liquid state followed by a cooling down to approximately 40°C. At the starting point of solidification, transmural left ventricular samples were inserted in vibratome plastic cassettes in which 2 transverse incisions were made at an interval of 4 mm. Luke warm agar was added to the samples and solidification was achieved after a few minutes. Consequently, a sharp blade was used to section the tissue at the level of the 2 incisions. After sectioning, approximately 3-4 mm thick samples were removed from the agar and placed in 0% calcium Normal Tyrode. A square block with a length and width of approximately 1.5 cm was cut from the mid-myocardial area of these sections for further analysis. Scar tissue was not embedded in agar since the thickness of the tissue already varied between 2 and 4 mm. Finally, samples were mounted on to the biaxial tissue tester (BioTester 5000, CellScale Biomaterials Testing, Waterloo, Canada) via a grip based attachment and submerged in 0% calcium Normal Tyrode in a temperature controlled bath heated at 37°C. Four surgical threads were glued on the surface of the samples. During biaxial stretch, loading pressure was limited to 20 kPa. 5 loading protocols were executed with ratio´s which differed in maximal loading in the x and y direction (x:y axis loading force ratios 1:1, 1:0.75, 1:0.5, 0.75:1, 0.5:1). Images and displacement magnitudes were acquired using a high resolution CCD camera to collect time synchronized images for analysis. Data points were analyzed according to the big deformation theory, mentioned in Sacks et al[39]. Over all change in the compliance was measured with a normalized maximal strain. Normalized maximal strain is defined as $\Omega_{nr} = (\lambda_1\lambda_2 - 1)/h$, where λ_i is the maximal principal strain in both directions, and h is the sample thickness.

We have used constitutive modeling to model the generalized response of the tissue. As the tissue structure of the MI scar is significantly different to the healthy LV tissue, we were limited in the usage of the microstructural and decided to use the well-known phenomenological Fung-type[40] model. A custom made routine, based on a least square data fitting, was used to fit the data to the chosen model.

According to this model, strain energy density function is given with:

$$W(Q) = \frac{1}{2} C_F (e^{Q} - 1)$$

$$Q = b_1 {E_{11}}^2 + b_2 {E_{22}}^2 + b_4 E_{11} E_{22}$$

Where C_F is material-like parameter, and b_i are non-dimensional parameters.

Based on which second Piola-Kirchoff stress is given with:

$$S_{11} = C_{iso} + C_F e^{Q} (b_1 E_{11} + b_4 E_{22})$$

$$S_{22} = C_{iso} + C_F e^{Q} (b_2 E_{22} + b_4 E_{11})$$

Average response per region was generated based on the average parameters for all of the available samples.

Supplemental figures and legends

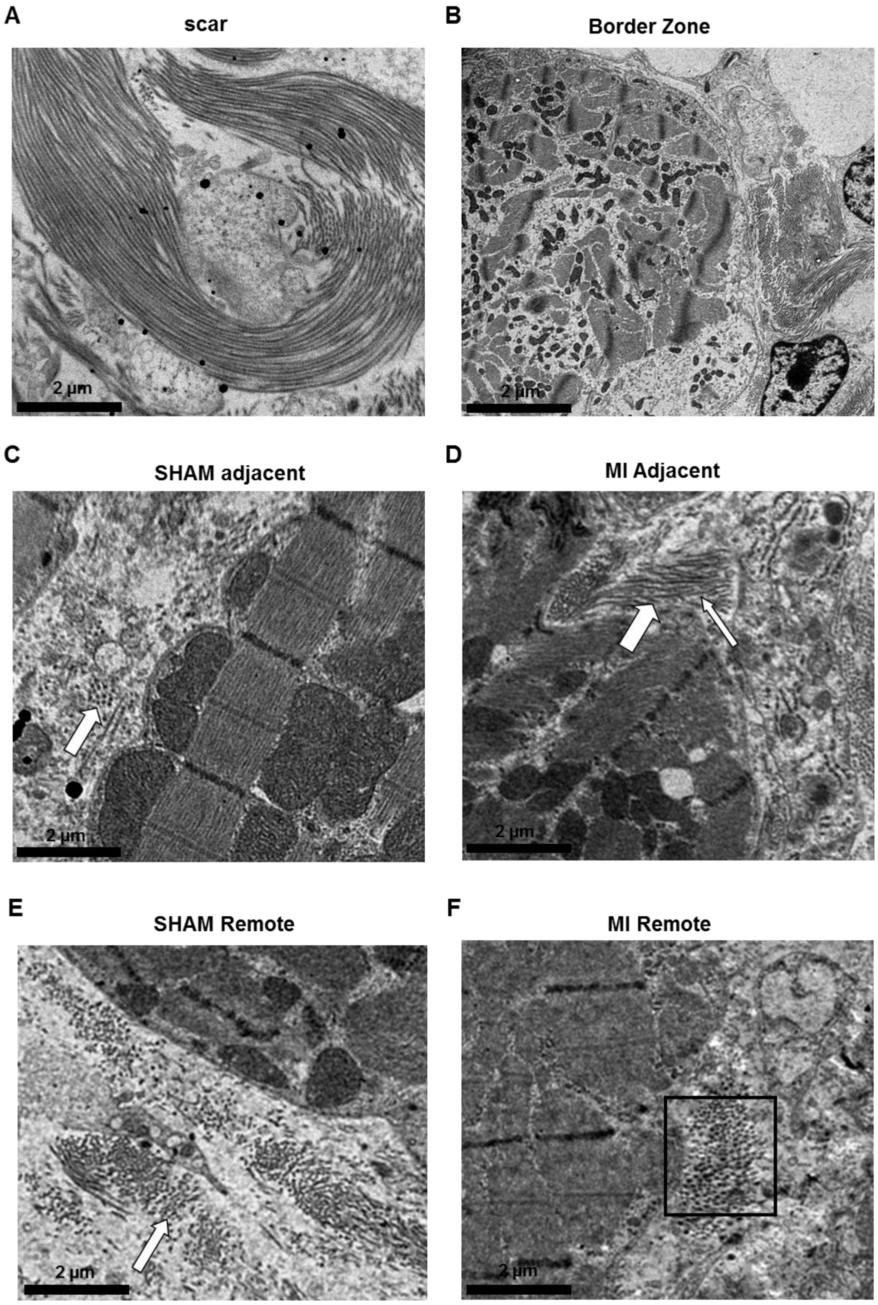

Supplemental Figure 1. Ultrastructural images of collagen fibers in the extracellular matrix of the adjacent and remote myocardium.

Cross-linking of collagen fibers in the scar (A), border zone (B, arrow), and adjacent myocardium (D, arrow). Loose collagen fibers in SHAM adjacent (C), SHAM remote (E) and MI remote (F). Scale bars represent 2 (A, B) and 5 µm (C-F). (SHAM n=3; MI n=4).

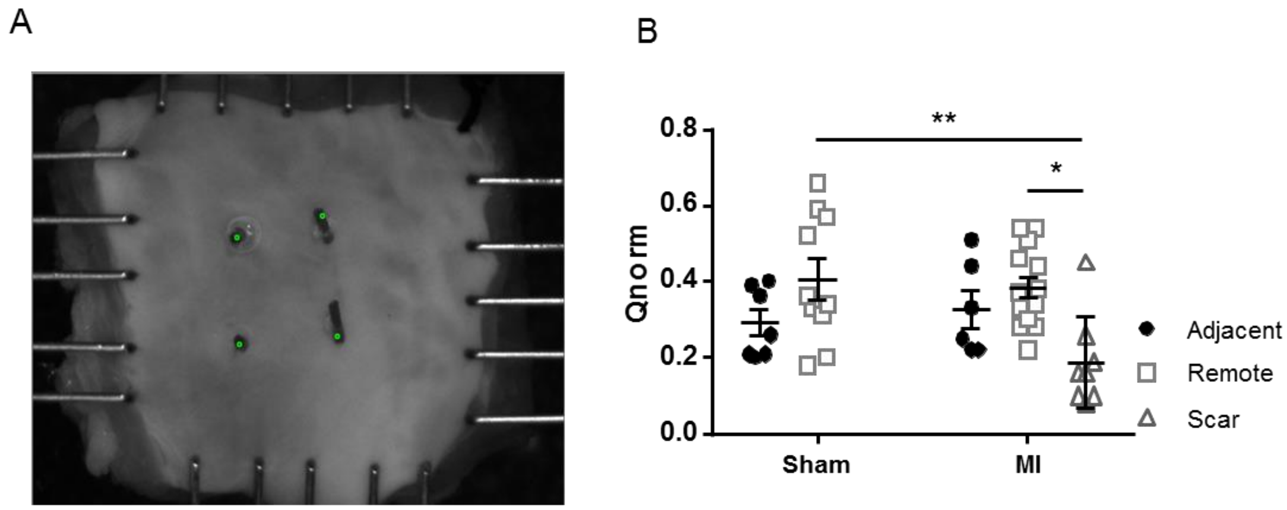

Supplemental Figure 2. Stiffness of the adjacent and remote myocardium.

(A) Representative image of bi-axial stretch of scar tissue. (B) Development of strain at 20 kPa and analysis of tissue compliance. Data are expressed as mean ± S.E.M. Data are expressed as mean ± S.E.M. (* $p<0.05$: ** $p<0.01$). (* $p<0.05$: ** $p<0.01$). (1-way ANOVA with Bonferroni post hoc test). Number of animals (SHAM n=7; MI n=13).

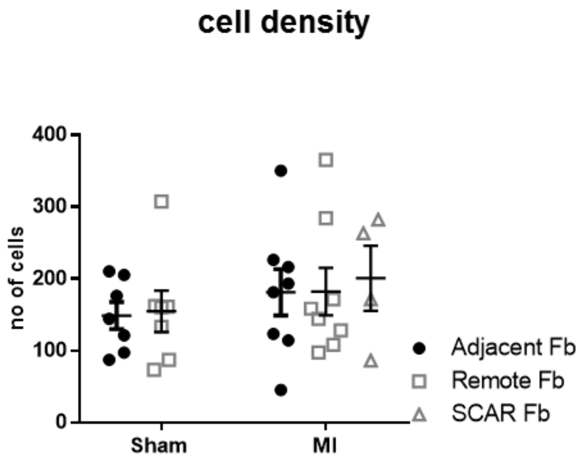

Supplemental Figure 3. Cell density in Fb cultures.

Quantification of number of cells after 4 days in culture from different regions in MI and SHAM. Cells are counted in 10 randomly chosen fields per dish for each group; total count is shown.

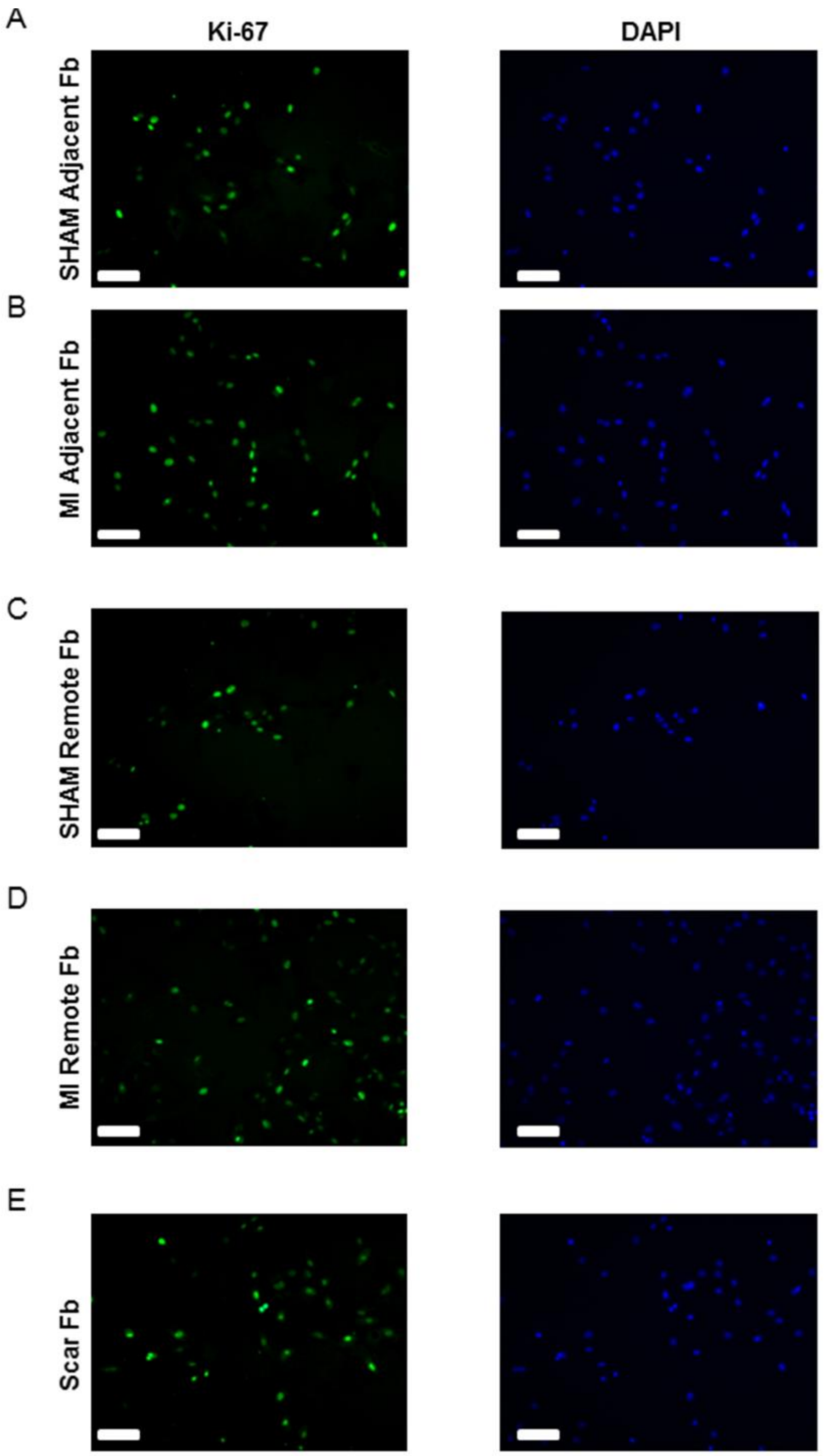

Supplemental Figure 4. Cell proliferation of fibroblast.

Immunofluorescent staining of the proliferation marker Ki-67 (green) and DAPI (blue) in Fb of SHAM and MI.

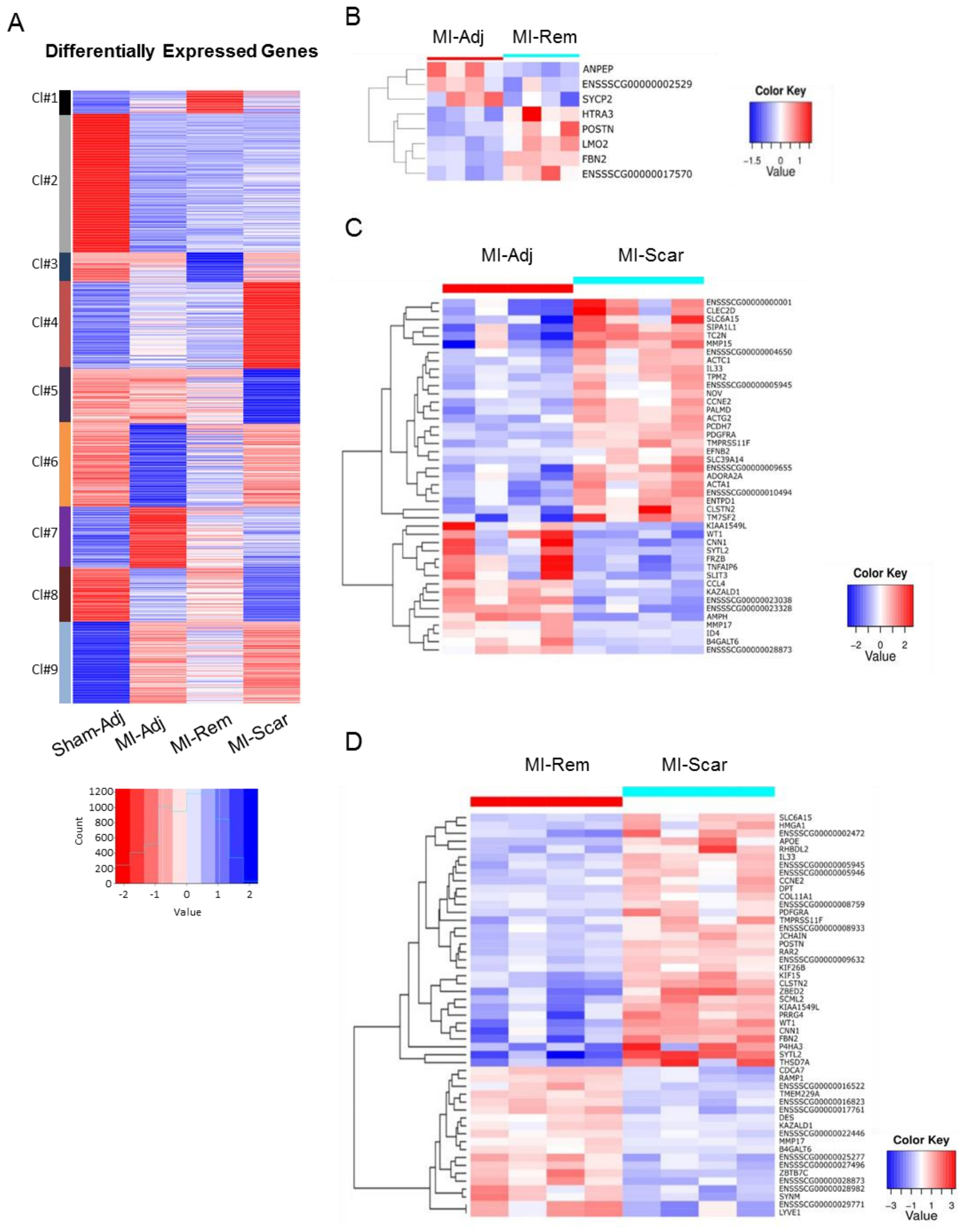

Supplemental Figure 5. Transcriptome analysis.

(A) Differential gene expression comparison clusters and potential signatures of Fb between SHAM (samples matched to adjacent region), $MI_{adjacent}$, MI_{remote} and scar; data are pooled per group. Differential gene expression heat map comparison including individual pig samples of Fb between $MI_{adjacent}$ and MI_{remote} (B); Scar and $MI_{adjacent}$ (C); and Scar and MI_{remote}(D).

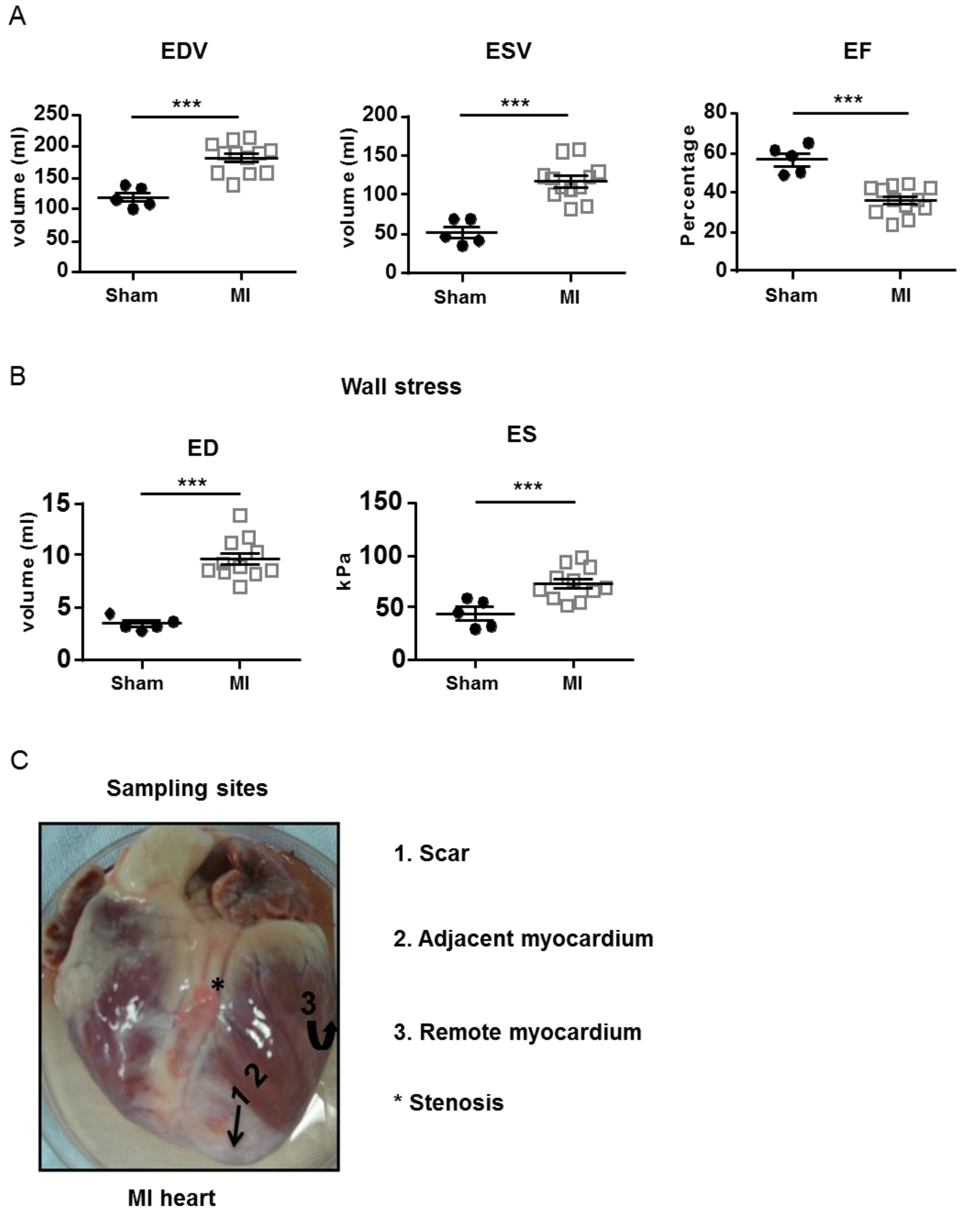

Supplemental Figure 6. Assessment of LV ventricular function 6 weeks after stent implantation.

End diastolic volume, end systolic volume and ejection fraction in LV of SHAM (n=5) and MI (n=12) animals (A). Increased EDP and ESP wall stress (B). Macroscopical image of myocardial infarct (MI) heart indicating tissue sampling sites (C). (*** p<0.01) (t-test unpaired)

Supplemental Table 1.

List of genes identified as cluster 1-9 through RNA sequencing in Supplementary Figure 5A. The full RNA-seq data will be publicly available for download from the Gene Expression Omnibus on publication (GSE98504).

Cluster 1

GeneID	Gene Name
ENSSSCG00000000660	A2M
ENSSSCG00000000883	
ENSSSCG00000002028	CMTM5
ENSSSCG00000004201	TMEM200A
ENSSSCG00000004452	PRSS35
ENSSSCG00000006692	
ENSSSCG00000007824	
ENSSSCG00000008348	
ENSSSCG00000008686	C4orf48
ENSSSCG00000008848	
ENSSSCG00000009004	SFRP2
ENSSSCG00000009526	ITGBL1
ENSSSCG00000012282	
ENSSSCG00000014118	
ENSSSCG00000014188	
ENSSSCG00000015125	
ENSSSCG00000016004	
ENSSSCG00000016336	
ENSSSCG00000016396	TNFAIP6
ENSSSCG00000019018	SNORA9
ENSSSCG00000019211	
ENSSSCG00000020152	SNORA48
ENSSSCG00000021114	U6
ENSSSCG00000021979	
ENSSSCG00000022427	RGS1
ENSSSCG00000022788	
ENSSSCG00000022872	
ENSSSCG00000023161	SNORA71
ENSSSCG00000023855	U3
ENSSSCG00000024662	
ENSSSCG00000024986	

ENSSSCG00000025190	
ENSSSCG00000025533	COX7A1
ENSSSCG00000026772	
ENSSSCG00000026938	
ENSSSCG00000027601	FAM180A
ENSSSCG00000027677	ABI3
ENSSSCG00000028966	U4
ENSSSCG00000029800	
ENSSSCG00000029961	
ENSSSCG00000030898	MAFB

Cluster 2

GeneID	Gene Name
ENSSSCG00000000551	ARNTL2
ENSSSCG00000000837	CHST11
ENSSSCG00000000914	BTG1
ENSSSCG00000000922	
ENSSSCG00000001061	JARID2
ENSSSCG00000001695	
ENSSSCG00000001873	
ENSSSCG00000001909	
ENSSSCG00000001963	EGLN3
ENSSSCG00000002141	
ENSSSCG00000002252	ARRDC4
ENSSSCG00000002294	ARG2
ENSSSCG00000002376	PGF
ENSSSCG00000002639	
ENSSSCG00000002826	
ENSSSCG00000003509	SH2D5
ENSSSCG00000003560	
ENSSSCG00000003586	EPB41
ENSSSCG00000003668	MYCL
ENSSSCG00000003725	
ENSSSCG00000003764	PTGFR
ENSSSCG00000003886	
ENSSSCG00000004195	
ENSSSCG00000004327	
ENSSSCG00000004345	
ENSSSCG00000004371	

ENSSSCG00000004473	
ENSSSCG00000004509	LIPG
ENSSSCG00000004531	C18orf54
ENSSSCG00000004601	MNS1
ENSSSCG00000004706	
ENSSSCG00000004769	
ENSSSCG00000005019	
ENSSSCG00000005206	MLANA
ENSSSCG00000005304	
ENSSSCG00000005423	
ENSSSCG00000005927	
ENSSSCG00000005981	FBXO32
ENSSSCG00000005992	SHAS2
ENSSSCG00000006053	
ENSSSCG00000006157	
ENSSSCG00000006198	
ENSSSCG00000006359	ADAMTS4
ENSSSCG00000006588	S100A9
ENSSSCG00000006589	S100A12
ENSSSCG00000006590	S100A8
ENSSSCG00000006987	SLC7A2
ENSSSCG00000007074	
ENSSSCG00000007140	SMOX
ENSSSCG00000007440	CD40
ENSSSCG00000007459	
ENSSSCG00000007687	
ENSSSCG00000007726	
ENSSSCG00000007888	TNFRSF17
ENSSSCG00000008084	
ENSSSCG00000008108	
ENSSSCG00000008203	IGKC
ENSSSCG00000008261	HK2
ENSSSCG00000008334	MXD1
ENSSSCG00000008427	KCNK12
ENSSSCG00000008443	EPAS1
ENSSSCG00000008535	CLIP4
ENSSSCG00000008648	RSAD2
ENSSSCG00000008716	
ENSSSCG00000008849	

ENSSSCG00000008930	TMPRSS11F
ENSSSCG00000009020	
ENSSSCG00000009082	
ENSSSCG00000009110	
ENSSSCG00000009637	
ENSSSCG00000009658	BNIP3L
ENSSSCG00000009701	
ENSSSCG00000009844	HSPB8
ENSSSCG00000009881	OAS2
ENSSSCG00000010037	
ENSSSCG00000010224	EGR2
ENSSSCG00000010292	P4HA1
ENSSSCG00000010381	
ENSSSCG00000010589	
ENSSSCG00000011043	C1QL3
ENSSSCG00000011264	CSRNP1
ENSSSCG00000011298	CDCP1
ENSSSCG00000011534	BHLHE40
ENSSSCG00000011648	
ENSSSCG00000011808	SST
ENSSSCG00000011945	
ENSSSCG00000011951	NFKBIZ
ENSSSCG00000012000	
ENSSSCG00000012170	PTCHD1
ENSSSCG00000012230	
ENSSSCG00000012356	SPIN3
ENSSSCG00000012481	TSPAN6
ENSSSCG00000012662	
ENSSSCG00000012829	
ENSSSCG00000012962	
ENSSSCG00000013102	MS4A7
ENSSSCG00000013366	LDHA
ENSSSCG00000013531	
ENSSSCG00000013735	
ENSSSCG00000013758	ZSWIM4
ENSSSCG00000014168	ELL2
ENSSSCG00000014431	AFAP1L1
ENSSSCG00000014878	PAK1
ENSSSCG00000014890	

ENSSSCG00000014974	
ENSSSCG00000014985	MMP1
ENSSSCG00000015014	ZC3H12C
ENSSSCG00000015045	NCAM1
ENSSSCG00000015407	GNAI1
ENSSSCG00000015446	
ENSSSCG00000015476	CHI3L1
ENSSSCG00000015487	TNFSF18
ENSSSCG00000015550	
ENSSSCG00000015595	ATF3
ENSSSCG00000015649	
ENSSSCG00000015664	CD55
ENSSSCG00000015688	CXCR4
ENSSSCG00000015791	
ENSSSCG00000015798	ANKRD37
ENSSSCG00000016230	EPHA4
ENSSSCG00000016231	
ENSSSCG00000016254	CCL20
ENSSSCG00000016633	MET
ENSSSCG00000016767	
ENSSSCG00000016887	ITGA2
ENSSSCG00000016937	
ENSSSCG00000017107	
ENSSSCG00000017110	
ENSSSCG00000017446	
ENSSSCG00000017574	
ENSSSCG00000018088	
ENSSSCG00000018357	SNORA19
ENSSSCG00000018620	SNORA17
ENSSSCG00000019619	SNORD10
ENSSSCG00000019898	U3
ENSSSCG00000020020	snoZ17
ENSSSCG00000020580	SNORA81
ENSSSCG00000020591	7SK
ENSSSCG00000020980	CH242-486P11.2
ENSSSCG00000021399	
ENSSSCG00000021559	
ENSSSCG00000021572	

ENSSSCG00000021720	
ENSSSCG00000021801	
ENSSSCG00000021816	
ENSSSCG00000021945	
ENSSSCG00000021967	SYK
ENSSSCG00000021980	
ENSSSCG00000022447	F3
ENSSSCG00000022789	
ENSSSCG00000022925	
ENSSSCG00000022977	
ENSSSCG00000023048	Metazoa_SRP
ENSSSCG00000023107	
ENSSSCG00000023221	
ENSSSCG00000023305	
ENSSSCG00000023420	
ENSSSCG00000023544	
ENSSSCG00000023684	
ENSSSCG00000023897	
ENSSSCG00000023953	SNORA23
ENSSSCG00000023957	CFAP126
ENSSSCG00000023967	
ENSSSCG00000024018	SLC16A3
ENSSSCG00000024207	
ENSSSCG00000024332	
ENSSSCG00000024596	NOCT
ENSSSCG00000024649	
ENSSSCG00000024720	
ENSSSCG00000025005	B4GALT6
ENSSSCG00000025181	
ENSSSCG00000025304	U3
ENSSSCG00000025378	
ENSSSCG00000025500	HTR1D
ENSSSCG00000025588	FJX1
ENSSSCG00000025726	
ENSSSCG00000025743	
ENSSSCG00000025744	KDSR
ENSSSCG00000025746	
ENSSSCG00000025783	ENTPD3
ENSSSCG00000025899	

ENSSSCG00000025956	
ENSSSCG00000025968	
ENSSSCG00000026026	
ENSSSCG00000026042	ULBP1
ENSSSCG00000026128	
ENSSSCG00000026159	
ENSSSCG00000026172	
ENSSSCG00000026173	ABCA1
ENSSSCG00000026249	
ENSSSCG00000026355	
ENSSSCG00000026693	
ENSSSCG00000026762	
ENSSSCG00000026780	
ENSSSCG00000026970	
ENSSSCG00000027005	
ENSSSCG00000027012	
ENSSSCG00000027158	CH242-209G14.2
ENSSSCG00000027199	
ENSSSCG00000027259	
ENSSSCG00000027354	
ENSSSCG00000027376	
ENSSSCG00000027438	
ENSSSCG00000027506	
ENSSSCG00000027643	
ENSSSCG00000027649	
ENSSSCG00000027868	
ENSSSCG00000027872	
ENSSSCG00000028006	
ENSSSCG00000028117	
ENSSSCG00000028322	BTG2
ENSSSCG00000028347	PPP1R11
ENSSSCG00000028351	
ENSSSCG00000028373	ENO2
ENSSSCG00000028461	CH242-240D14.1
ENSSSCG00000028511	
ENSSSCG00000028524	
ENSSSCG00000028586	

ENSSSCG00000028736	
ENSSSCG00000028852	
ENSSSCG00000028882	
ENSSSCG00000028977	
ENSSSCG00000029030	
ENSSSCG00000029033	
ENSSSCG00000029075	
ENSSSCG00000029214	
ENSSSCG00000029230	ECM1
ENSSSCG00000029384	
ENSSSCG00000029446	
ENSSSCG00000029603	
ENSSSCG00000029621	BMPR1B
ENSSSCG00000029640	
ENSSSCG00000029705	
ENSSSCG00000029736	
ENSSSCG00000030057	
ENSSSCG00000030262	GDPD1
ENSSSCG00000030282	
ENSSSCG00000030323	
ENSSSCG00000030410	
ENSSSCG00000030477	SNORA61
ENSSSCG00000030540	
ENSSSCG00000030570	
ENSSSCG00000030596	HES2
ENSSSCG00000030781	C17orf67
ENSSSCG00000030859	CH242-277I8.1
ENSSSCG00000030887	CH242-257M5.2
ENSSSCG00000030931	CU302278.9
ENSSSCG00000031039	CH242-138G12.1

Cluster 3

Gene ID	GeneName
ENSSSCG00000000647	OLR1
ENSSSCG00000002410	
ENSSSCG00000006862	VCAM1
ENSSSCG00000007189	SDCBP2

ENSSSCG00000008712	
ENSSSCG00000008954	
ENSSSCG00000009024	
ENSSSCG00000011019	
ENSSSCG00000011702	TM4SF1
ENSSSCG00000013749	
ENSSSCG00000014138	
ENSSSCG00000014255	SLC12A2
ENSSSCG00000015640	
ENSSSCG00000016729	
ENSSSCG00000017570	
ENSSSCG00000017576	
ENSSSCG00000018104	SNORA19
ENSSSCG00000018196	SNORD57
ENSSSCG00000018328	
ENSSSCG00000018378	snoU13
ENSSSCG00000018402	SNORA46
ENSSSCG00000018584	snosnR61
ENSSSCG00000018715	Y_RNA
ENSSSCG00000018864	SNORD89
ENSSSCG00000018891	SNORA74
ENSSSCG00000019023	5S_rRNA
ENSSSCG00000019316	5S_rRNA
ENSSSCG00000019334	
ENSSSCG00000019755	SNORD77
ENSSSCG00000019783	SNORD42
ENSSSCG00000019809	SNORD18
ENSSSCG00000021104	
ENSSSCG00000022944	HIST2H2BE
ENSSSCG00000023798	SNORD88
ENSSSCG00000024239	
ENSSSCG00000026049	
ENSSSCG00000026212	SNORD29
ENSSSCG00000026215	
ENSSSCG00000026445	
ENSSSCG00000026506	
ENSSSCG00000026834	
ENSSSCG00000026850	
ENSSSCG00000027280	SNORD99

ENSSSCG00000027820	CD200
ENSSSCG00000027879	
ENSSSCG00000028235	
ENSSSCG00000028949	
ENSSSCG00000029372	
ENSSSCG00000029381	
ENSSSCG00000029683	
ENSSSCG00000030102	SNORA26
ENSSSCG00000030624	
ENSSSCG00000031003	CH242-442M8.1

Cluster 4

Gene ID	Gene Name
ENSSSCG00000000002	GTSE1
ENSSSCG00000000010	FBLN1
ENSSSCG00000000408	
ENSSSCG00000000471	
ENSSSCG00000000683	CDCA3
ENSSSCG00000000693	
ENSSSCG00000000739	FOXM1
ENSSSCG00000000751	CCDC77
ENSSSCG00000000915	DCN
ENSSSCG00000001134	
ENSSSCG00000001239	
ENSSSCG00000001500	
ENSSSCG00000001526	HMGA1
ENSSSCG00000001816	
ENSSSCG00000002663	GINS2
ENSSSCG00000002684	
ENSSSCG00000002754	NQO1
ENSSSCG00000002768	CENPT
ENSSSCG00000002998	SNRPA
ENSSSCG00000003919	MUTYH
ENSSSCG00000004027	PDE10A
ENSSSCG00000004579	
ENSSSCG00000005071	C14orf37
ENSSSCG00000005311	CD72
ENSSSCG00000005533	PTGS1

ENSSSCG00000006035	ANGPT1
ENSSSCG00000006243	
ENSSSCG00000006273	MCM4
ENSSSCG00000006296	ATP1B1
ENSSSCG00000006297	DPT
ENSSSCG00000006832	PSRC1
ENSSSCG00000006911	
ENSSSCG00000007006	
ENSSSCG00000007151	CDC25B
ENSSSCG00000007268	E2F1
ENSSSCG00000007391	MATN4
ENSSSCG00000008010	CHTF18
ENSSSCG00000008446	SIX2
ENSSSCG00000008579	CENPO
ENSSSCG00000008623	
ENSSSCG00000008729	LYAR
ENSSSCG00000008841	PDGFRA
ENSSSCG00000008882	
ENSSSCG00000008957	AMCF-II
ENSSSCG00000009009	MND1
ENSSSCG00000009094	CCNA2
ENSSSCG00000009671	PBK
ENSSSCG00000009704	HMGB2
ENSSSCG00000009938	UNG
ENSSSCG00000010561	TLX1
ENSSSCG00000010991	AQP3
ENSSSCG00000011129	
ENSSSCG00000011454	SFMBT1
ENSSSCG00000011624	MCM2
ENSSSCG00000011641	
ENSSSCG00000011673	
ENSSSCG00000011722	RAP2B
ENSSSCG00000012056	CHAF1B
ENSSSCG00000012135	VEGFD
ENSSSCG00000012871	FGF19
ENSSSCG00000012967	FOSL1
ENSSSCG00000013008	CDCA5
ENSSSCG00000013181	SERPING1
ENSSSCG00000013387	

ENSSSCG00000013408	ADM
ENSSSCG00000013509	CHAF1A
ENSSSCG00000013517	UHRF1
ENSSSCG00000013599	ANGPTL4
ENSSSCG00000013772	ASF1B
ENSSSCG00000013904	
ENSSSCG00000014248	LMNB1
ENSSSCG00000014256	FBN2
ENSSSCG00000014268	
ENSSSCG00000014326	KIF20A
ENSSSCG00000015022	LAYN
ENSSSCG00000015413	FGL2
ENSSSCG00000015453	PDIA4
ENSSSCG00000015604	NEK2
ENSSSCG00000015961	CDCA7
ENSSSCG00000016063	
ENSSSCG00000016258	
ENSSSCG00000016427	XRCC2
ENSSSCG00000016650	
ENSSSCG00000016841	SLC1A3
ENSSSCG00000016886	
ENSSSCG00000016892	FST
ENSSSCG00000017256	
ENSSSCG00000017396	PSMC3IP
ENSSSCG00000017408	STAT5A
ENSSSCG00000017471	CDC6
ENSSSCG00000017758	SPAG5
ENSSSCG00000017893	FAM64A
ENSSSCG00000017904	ENO3
ENSSSCG00000018064	
ENSSSCG00000018172	SNORA63
ENSSSCG00000018225	SNORA18
ENSSSCG00000018372	SNORD86
ENSSSCG00000018972	SNORA33
ENSSSCG00000019418	SNORA4
ENSSSCG00000019894	SNORA62
ENSSSCG00000020533	SNORA2
ENSSSCG00000021576	CD83
ENSSSCG00000021585	OXTR

ENSSSCG00000021608	
ENSSSCG00000021826	
ENSSSCG00000021875	SNORA70
ENSSSCG00000021983	5S_rRNA
ENSSSCG00000022092	
ENSSSCG00000022429	KAZALD1
ENSSSCG00000022983	
ENSSSCG00000023038	
ENSSSCG00000023069	
ENSSSCG00000023143	SNORD34
ENSSSCG00000023176	TROAP
ENSSSCG00000023440	
ENSSSCG00000023482	
ENSSSCG00000023529	
ENSSSCG00000023716	
ENSSSCG00000024660	
ENSSSCG00000024852	U3
ENSSSCG00000024997	
ENSSSCG00000025066	
ENSSSCG00000025277	
ENSSSCG00000025488	MCM3
ENSSSCG00000025513	SNORD22
ENSSSCG00000025761	
ENSSSCG00000025971	
ENSSSCG00000026041	
ENSSSCG00000026055	
ENSSSCG00000026064	LSM5
ENSSSCG00000026265	
ENSSSCG00000026426	SNORA70
ENSSSCG00000026558	
ENSSSCG00000026575	
ENSSSCG00000026608	
ENSSSCG00000026668	
ENSSSCG00000026951	
ENSSSCG00000027364	
ENSSSCG00000027582	
ENSSSCG00000027665	SIRPB1
ENSSSCG00000028063	TACC2
ENSSSCG00000028202	

ENSSSCG00000028240	
ENSSSCG00000028301	
ENSSSCG00000028695	
ENSSSCG00000028696	FEN1
ENSSSCG00000028793	
ENSSSCG00000028873	
ENSSSCG00000028924	AURKB
ENSSSCG00000028982	
ENSSSCG00000029481	CPLX1
ENSSSCG00000029509	KIF22
ENSSSCG00000030156	
ENSSSCG00000030580	
ENSSSCG00000030921	APOA1

Cluster 5

Gene ID	Gene Name
ENSSSCG00000000602	RERG
ENSSSCG00000000763	
ENSSSCG00000001689	
ENSSSCG00000002383	FOS
ENSSSCG00000002385	TGFB3
ENSSSCG00000002806	MMP15
ENSSSCG00000002853	
ENSSSCG00000003521	
ENSSSCG00000003644	
ENSSSCG00000003922	UROD
ENSSSCG00000004052	FNDC1
ENSSSCG00000004475	HTR1B
ENSSSCG00000004803	ACTC1
ENSSSCG00000004961	ITGA11
ENSSSCG00000005278	PRUNE2
ENSSSCG00000005316	TPM2
ENSSSCG00000005945	
ENSSSCG00000005946	
ENSSSCG00000006321	FAM78B
ENSSSCG00000006472	CRABP2
ENSSSCG00000006857	COL11A1
ENSSSCG00000008072	ASPN
ENSSSCG00000008205	

ENSSSCG00000008294	ACTG2
ENSSSCG00000008340	ANTXR1
ENSSSCG00000008813	CORIN
ENSSSCG00000009446	PCDH17
ENSSSCG00000009523	GGACT
ENSSSCG00000009580	S1PR3
ENSSSCG00000009638	
ENSSSCG00000010370	ANXA8
ENSSSCG00000010437	PAPSS2
ENSSSCG00000010497	ENTPD1
ENSSSCG00000010698	
ENSSSCG00000010728	CPXM2
ENSSSCG00000010879	KIF26B
ENSSSCG00000011046	ITGA8
ENSSSCG00000011110	CCDC3
ENSSSCG00000011973	COL8A1
ENSSSCG00000012561	FRMPD3
ENSSSCG00000013316	WT1
ENSSSCG00000013614	CNN1
ENSSSCG00000014840	P4HA3
ENSSSCG00000014909	SYTL2
ENSSSCG00000014980	C11orf70
ENSSSCG00000015763	
ENSSSCG00000016002	
ENSSSCG00000016222	KCNE4
ENSSSCG00000016522	
ENSSSCG00000016691	JAZF1
ENSSSCG00000016741	
ENSSSCG00000016784	ANKH
ENSSSCG00000016823	
ENSSSCG00000017717	UNC45B
ENSSSCG00000017761	UNC119
ENSSSCG00000018001	
ENSSSCG00000018062	
ENSSSCG00000018101	Y_RNA
ENSSSCG00000018184	snoU83B
ENSSSCG00000018466	
ENSSSCG00000018563	SNORA48
ENSSSCG00000018869	5S_rRNA

ENSSSCG00000019364	ssc-let-7d
ENSSSCG00000019944	SNORD97
ENSSSCG00000020785	DES
ENSSSCG00000020958	
ENSSSCG00000021240	
ENSSSCG00000021356	
ENSSSCG00000021387	
ENSSSCG00000021518	
ENSSSCG00000022211	U5
ENSSSCG00000022254	
ENSSSCG00000022288	STK38L
ENSSSCG00000022913	SLPI
ENSSSCG00000022959	
ENSSSCG00000023065	
ENSSSCG00000024236	MMP17
ENSSSCG00000024495	SELPLG
ENSSSCG00000024521	SNORD35
ENSSSCG00000025858	
ENSSSCG00000025924	IGFBP5
ENSSSCG00000026100	
ENSSSCG00000026628	
ENSSSCG00000027055	NUAK1
ENSSSCG00000027204	
ENSSSCG00000027487	LAT2
ENSSSCG00000027496	
ENSSSCG00000029421	
ENSSSCG00000029613	SYNM
ENSSSCG00000029998	KLF7
ENSSSCG00000030345	INPP5J
ENSSSCG00000030387	KIAA0040
ENSSSCG00000030418	
ENSSSCG00000030575	TMEM158
ENSSSCG00000030699	
ENSSSCG00000030825	IGLV-9
ENSSSCG00000030868	IGLV-10
ENSSSCG00000031037	IGLV-7

Cluster 6

Gene ID	Genen Name

ENSSSCG00000000060	DESI1
ENSSSCG00000000181	RND1
ENSSSCG00000000242	CH242-185F9.2
ENSSSCG00000000246	
ENSSSCG00000000475	IRAK3
ENSSSCG00000000504	PTPRB
ENSSSCG00000000544	PTHLH
ENSSSCG00000000657	CLEC2D
ENSSSCG00000001397	TMP-CH242-74M17.4
ENSSSCG00000001404	TNF
ENSSSCG00000001952	NFKBIA
ENSSSCG00000002135	PNP
ENSSSCG00000003079	PVR
ENSSSCG00000003451	
ENSSSCG00000003669	MFSD2A
ENSSSCG00000003744	
ENSSSCG00000003805	PDE4B
ENSSSCG00000003839	PLPP3
ENSSSCG00000004121	FBXO30
ENSSSCG00000004154	TNFAIP3
ENSSSCG00000004466	TTK
ENSSSCG00000004793	SPRED1
ENSSSCG00000004890	SERPINB2
ENSSSCG00000005037	ERO1A
ENSSSCG00000005203	IL33
ENSSSCG00000005403	
ENSSSCG00000005494	
ENSSSCG00000005688	PTGES
ENSSSCG00000005944	NDRG1
ENSSSCG00000006334	RGS5
ENSSSCG00000006351	PCP4L1
ENSSSCG00000006747	
ENSSSCG00000006891	GCLM
ENSSSCG00000006917	LRRC8C
ENSSSCG00000007007	IDO1
ENSSSCG00000007331	RBL1
ENSSSCG00000007554	ZFAND2A
ENSSSCG00000007793	

ENSSSCG00000008088	IL1B1
ENSSSCG00000008090	IL1A
ENSSSCG00000008311	CYP26B1
ENSSSCG00000008953	CXCL8
ENSSSCG00000008959	CXCL2
ENSSSCG00000008963	AREG
ENSSSCG00000009409	
ENSSSCG00000009448	
ENSSSCG00000009623	SLC39A14
ENSSSCG00000009837	
ENSSSCG00000009865	TBX3
ENSSSCG00000009996	LIF
ENSSSCG00000010054	ADORA2A
ENSSSCG00000010103	
ENSSSCG00000010302	USP54
ENSSSCG00000010338	DYDC1
ENSSSCG00000010340	FAM213A
ENSSSCG00000011133	PFKFB3
ENSSSCG00000011218	SLC4A7
ENSSSCG00000011683	PCOLCE2
ENSSSCG00000011727	PTX3
ENSSSCG00000011848	TFRC
ENSSSCG00000011925	CD200R1
ENSSSCG00000012051	RUNX1
ENSSSCG00000012277	TIMP1
ENSSSCG00000012583	
ENSSSCG00000012812	CH242-305A15.4
ENSSSCG00000013297	CD44
ENSSSCG00000013655	ICAM1
ENSSSCG00000014249	MARCH3
ENSSSCG00000015293	
ENSSSCG00000015299	STEAP4
ENSSSCG00000015579	PTGS2
ENSSSCG00000015581	CENPF
ENSSSCG00000015770	VEGFC
ENSSSCG00000016101	CFLAR
ENSSSCG00000016308	
ENSSSCG00000016322	ACKR3
ENSSSCG00000016900	

ENSSSCG00000017091	TNIP1
ENSSSCG00000017698	CCL4
ENSSSCG00000017700	CCL3L1
ENSSSCG00000017702	CCL23
ENSSSCG00000017705	CCL5
ENSSSCG00000017721	CCL8
ENSSSCG00000017722	CCL11
ENSSSCG00000017723	CCL2
ENSSSCG00000018070	
ENSSSCG00000018085	
ENSSSCG00000018095	
ENSSSCG00000018329	SNORA1
ENSSSCG00000018333	SNORD79
ENSSSCG00000018450	SNORD100
ENSSSCG00000018461	U1
ENSSSCG00000018462	SNORD65
ENSSSCG00000018580	SNORA65
ENSSSCG00000018923	SNORD21
ENSSSCG00000018964	SNORA67
ENSSSCG00000019069	SNORD78
ENSSSCG00000019430	
ENSSSCG00000019599	SNORA51
ENSSSCG00000019699	SNORD45
ENSSSCG00000019707	SNORA75
ENSSSCG00000020211	U6
ENSSSCG00000020213	
ENSSSCG00000020705	MAP3K8
ENSSSCG00000020721	
ENSSSCG00000020906	TNFSF10
ENSSSCG00000020970	IL-6
ENSSSCG00000021027	
ENSSSCG00000021206	IL1RAP
ENSSSCG00000021687	
ENSSSCG00000021723	
ENSSSCG00000022742	PRDX6
ENSSSCG00000022961	
ENSSSCG00000023095	
ENSSSCG00000023466	SNORD14
ENSSSCG00000023504	

ENSSSCG00000023737	CSF2
ENSSSCG00000023796	IL18BP
ENSSSCG00000024080	
ENSSSCG00000024853	ssc-mir-4332
ENSSSCG00000024933	SLC36A4
ENSSSCG00000025206	RNF19B
ENSSSCG00000025715	SNORD15
ENSSSCG00000025844	
ENSSSCG00000025922	
ENSSSCG00000026040	
ENSSSCG00000026391	
ENSSSCG00000026454	
ENSSSCG00000026492	
ENSSSCG00000026602	PTGIR
ENSSSCG00000027030	BDKRB2
ENSSSCG00000027521	
ENSSSCG00000027607	IER3
ENSSSCG00000027624	SNORD14
ENSSSCG00000027646	TIPARP
ENSSSCG00000027850	IL6
ENSSSCG00000028329	SNORD58
ENSSSCG00000028483	SNORD33
ENSSSCG00000028484	CLDN1
ENSSSCG00000028525	
ENSSSCG00000028604	PRCD
ENSSSCG00000028605	
ENSSSCG00000029095	SNORD28
ENSSSCG00000029605	Metazoa_SRP
ENSSSCG00000029756	ADGRG2
ENSSSCG00000029876	SOD2
ENSSSCG00000030165	MAFF
ENSSSCG00000030278	MLLT11
ENSSSCG00000030300	MT2A

Cluster 7

Gene ID	Gene Name
ENSSSCG00000000606	MGP
ENSSSCG00000000710	
ENSSSCG00000001620	MDFI

ENSSSCG00000002511	
ENSSSCG00000002557	NUDT14
ENSSSCG00000002898	
ENSSSCG00000002922	THAP8
ENSSSCG00000003439	DHRS3
ENSSSCG00000003479	MFAP2
ENSSSCG00000003693	MYOM1
ENSSSCG00000003705	CABLES1
ENSSSCG00000003974	CITED4
ENSSSCG00000004012	THBS2
ENSSSCG00000004013	
ENSSSCG00000004248	PLN
ENSSSCG00000004444	FAM26F
ENSSSCG00000004468	SH3BGRL2
ENSSSCG00000005481	
ENSSSCG00000006002	NOV
ENSSSCG00000006051	
ENSSSCG00000006874	PALMD
ENSSSCG00000007100	
ENSSSCG00000007115	THBD
ENSSSCG00000007369	
ENSSSCG00000007541	PDGFA
ENSSSCG00000008430	
ENSSSCG00000008528	LBH
ENSSSCG00000008606	
ENSSSCG00000008765	PCDH7
ENSSSCG00000008843	
ENSSSCG00000009111	SYNPO2
ENSSSCG00000009304	
ENSSSCG00000009565	GAS6
ENSSSCG00000009625	
ENSSSCG00000009703	HAND2
ENSSSCG00000009745	ADGRD1
ENSSSCG00000009931	MVK
ENSSSCG00000009947	CMKLR1
ENSSSCG00000010190	ACTA1
ENSSSCG00000010209	FAM13C
ENSSSCG00000010447	ACTA2
ENSSSCG00000010593	CNNM2

ENSSSCG00000010816	TGFB2
ENSSSCG00000010965	CCL21
ENSSSCG00000011014	BAMBI
ENSSSCG00000011326	PTH1R
ENSSSCG00000011379	KLHDC8B
ENSSSCG00000011478	FAM107A
ENSSSCG00000011664	RBP1
ENSSSCG00000012028	
ENSSSCG00000012049	
ENSSSCG00000012490	TMEM35A
ENSSSCG00000012792	RENBP
ENSSSCG00000013418	CFD
ENSSSCG00000013590	CTXN1
ENSSSCG00000014131	SSBP2
ENSSSCG00000014149	MEF2C
ENSSSCG00000014157	NR2F1
ENSSSCG00000015015	ARHGAP20
ENSSSCG00000015070	APOA1
ENSSSCG00000015116	MCAM
ENSSSCG00000015797	
ENSSSCG00000015862	LIMS2
ENSSSCG00000016290	EFHD1
ENSSSCG00000016435	SMARCD3
ENSSSCG00000016437	
ENSSSCG00000017041	ADRA1B
ENSSSCG00000017340	DCAKD
ENSSSCG00000017472	IGFBP4
ENSSSCG00000017971	NAA38
ENSSSCG00000017987	MYH10
ENSSSCG00000018178	SNORA40
ENSSSCG00000020720	MRVI1
ENSSSCG00000020821	
ENSSSCG00000021280	
ENSSSCG00000021283	
ENSSSCG00000021374	FXYD1
ENSSSCG00000021598	EVA1C
ENSSSCG00000021685	
ENSSSCG00000021736	
ENSSSCG00000021880	MXRA8

ENSSSCG00000022280	DACT3
ENSSSCG00000022998	PKIG
ENSSSCG00000023498	
ENSSSCG00000024230	MYL9
ENSSSCG00000024515	SNORD14
ENSSSCG00000024644	CIDEA
ENSSSCG00000024651	HEYL
ENSSSCG00000024708	
ENSSSCG00000024936	C1QTNF5
ENSSSCG00000025176	
ENSSSCG00000026208	
ENSSSCG00000026463	
ENSSSCG00000026757	
ENSSSCG00000027124	
ENSSSCG00000027157	
ENSSSCG00000027297	SNORD14
ENSSSCG00000027341	
ENSSSCG00000027471	SNORA54
ENSSSCG00000027762	CU633166.1
ENSSSCG00000027928	TMEM9
ENSSSCG00000028421	
ENSSSCG00000028831	
ENSSSCG00000029549	SNORD22
ENSSSCG00000029722	
ENSSSCG00000029791	
ENSSSCG00000030048	PLEKHG2
ENSSSCG00000030160	
ENSSSCG00000030358	

Cluster 8

Gene ID	Gene Name
ENSSSCG00000000420	
ENSSSCG00000000519	GLIPR1
ENSSSCG00000000951	CSRP2
ENSSSCG00000001408	AIF1
ENSSSCG00000001694	
ENSSSCG00000001841	RHCG
ENSSSCG00000001849	ANPEP
ENSSSCG00000002471	ISG12(A)

ENSSSCG00000002551	
ENSSSCG00000003063	PHLDB3
ENSSSCG00000003080	IGSF23
ENSSSCG00000003582	SMPDL3B
ENSSSCG00000003651	RHBDL2
ENSSSCG00000003722	CDH2
ENSSSCG00000003846	GLIS1
ENSSSCG00000004565	CA12
ENSSSCG00000005721	
ENSSSCG00000006052	FZD6
ENSSSCG00000006197	SULF1
ENSSSCG00000006235	TOX
ENSSSCG00000006647	CTSK
ENSSSCG00000006651	ADAMTSL4
ENSSSCG00000007436	MMP9
ENSSSCG00000007482	TSHZ2
ENSSSCG00000007897	
ENSSSCG00000008101	
ENSSSCG00000008356	
ENSSSCG00000008888	NPY1R
ENSSSCG00000008933	
ENSSSCG00000009003	
ENSSSCG00000009138	CFI
ENSSSCG00000009361	POSTN
ENSSSCG00000009489	
ENSSSCG00000009593	ROR2
ENSSSCG00000009635	
ENSSSCG00000010044	IGLC
ENSSSCG00000010746	ADAM12
ENSSSCG00000011355	COL7A1
ENSSSCG00000011495	PRICKLE2
ENSSSCG00000011689	PLOD2
ENSSSCG00000011877	CD86
ENSSSCG00000011936	ZBED2
ENSSSCG00000011946	ALCAM
ENSSSCG00000012151	NHS
ENSSSCG00000013400	
ENSSSCG00000013940	NLRP3
ENSSSCG00000014310	CXCL14

ENSSSCG00000014316	TGFBI
ENSSSCG00000015025	CRYAB
ENSSSCG00000015520	
ENSSSCG00000015580	
ENSSSCG00000015965	GPR155
ENSSSCG00000016174	FN1
ENSSSCG00000016331	RAMP1
ENSSSCG00000016443	TMEM176A
ENSSSCG00000017759	ALDOC
ENSSSCG00000018605	SNORA20
ENSSSCG00000018942	SNORD77
ENSSSCG00000019323	SNORA13
ENSSSCG00000019612	SNORD24
ENSSSCG00000020731	
ENSSSCG00000020864	VDR
ENSSSCG00000021068	TRAF5
ENSSSCG00000021722	
ENSSSCG00000021747	
ENSSSCG00000021777	
ENSSSCG00000021865	INHBA
ENSSSCG00000022232	
ENSSSCG00000022818	
ENSSSCG00000023085	STAC
ENSSSCG00000023606	
ENSSSCG00000023742	
ENSSSCG00000023770	
ENSSSCG00000023773	ARG1
ENSSSCG00000024022	TRPC6
ENSSSCG00000024132	TMEM47
ENSSSCG00000024158	
ENSSSCG00000024669	
ENSSSCG00000024806	
ENSSSCG00000026384	
ENSSSCG00000027777	PRRG1
ENSSSCG00000027935	FHOD3
ENSSSCG00000027955	CAMK1D
ENSSSCG00000028076	ZBTB7C
ENSSSCG00000028253	
ENSSSCG00000029049	

ENSSSCG00000029076	
ENSSSCG00000029358	
ENSSSCG00000029783	MKX
ENSSSCG00000030461	
ENSSSCG00000030680	TCF7
ENSSSCG00000030775	IGLV-11
ENSSSCG00000030789	IGLV-12
ENSSSCG00000030998	
ENSSSCG00000031023	
ENSSSCG00000031054	IGLV-8

Cluster 9

Gene ID	Gene Name
ENSSSCG00000000133	TST
ENSSSCG00000000975	PANX2
ENSSSCG00000000981	CRELD2
ENSSSCG00000001198	
ENSSSCG00000001244	
ENSSSCG00000001783	
ENSSSCG00000001860	NRG4
ENSSSCG00000001906	CYP1A1
ENSSSCG00000001910	ISLR
ENSSSCG00000002515	SLC25A47
ENSSSCG00000002866	CEBPA
ENSSSCG00000003333	C1QTNF12
ENSSSCG00000003527	EPHB2
ENSSSCG00000003876	CDKN2C
ENSSSCG00000003949	CDC20
ENSSSCG00000004049	ACAT2
ENSSSCG00000004174	TCF21
ENSSSCG00000004633	TNFAIP8L3
ENSSSCG00000004672	GATM
ENSSSCG00000004875	CYB5A
ENSSSCG00000004907	CCBE1
ENSSSCG00000004948	SMAD6
ENSSSCG00000005045	BMP4
ENSSSCG00000005190	NFIB
ENSSSCG00000005443	CTNNAL1
ENSSSCG00000005455	SVEP1

ENSSSCG00000005967	FAM84B
ENSSSCG00000006082	
ENSSSCG00000006245	SDR16C5
ENSSSCG00000006474	
ENSSSCG00000006821	GSTM3
ENSSSCG00000006924	GBP1
ENSSSCG00000007073	ISM1
ENSSSCG00000007227	ID1
ENSSSCG00000007344	KIAA1755
ENSSSCG00000007528	PHACTR3
ENSSSCG00000007572	LFNG
ENSSSCG00000007586	FSCN1
ENSSSCG00000008142	C2orf40
ENSSSCG00000008230	ATOH8
ENSSSCG00000008397	EFEMP1
ENSSSCG00000008723	HTRA3
ENSSSCG00000008737	C1QTNF7
ENSSSCG00000008835	RASL11B
ENSSSCG00000009083	SPRY1
ENSSSCG00000009132	ENPEP
ENSSSCG00000009222	SPARCL1
ENSSSCG00000010085	SDF2L1
ENSSSCG00000010241	
ENSSSCG00000010461	ANKRD1
ENSSSCG00000010966	CCL19
ENSSSCG00000011184	
ENSSSCG00000011393	UBA7
ENSSSCG00000012136	PIR
ENSSSCG00000012257	MAOA
ENSSSCG00000012680	GPC3
ENSSSCG00000012758	NSDHL
ENSSSCG00000012873	
ENSSSCG00000012902	CDK2AP2
ENSSSCG00000013302	CAT
ENSSSCG00000013896	MPV17L2
ENSSSCG00000014041	MXD3
ENSSSCG00000014219	CDO1
ENSSSCG00000014920	FZD4
ENSSSCG00000014971	

ENSSSCG00000015357	MEOX2
ENSSSCG00000015563	RGL1
ENSSSCG00000015622	CD34
ENSSSCG00000015724	
ENSSSCG00000015796	PDLIM3
ENSSSCG00000016373	
ENSSSCG00000016386	
ENSSSCG00000016758	STK17A
ENSSSCG00000016872	HMGCS1
ENSSSCG00000017199	TRIM47
ENSSSCG00000017257	
ENSSSCG00000017277	PECAM1
ENSSSCG00000017307	MYL4
ENSSSCG00000017380	ARL4D
ENSSSCG00000017583	SGCA
ENSSSCG00000017753	KSR1
ENSSSCG00000017874	
ENSSSCG00000018758	ssc-mir-214
ENSSSCG00000019093	SNORD16
ENSSSCG00000019135	SNORD50
ENSSSCG00000019142	
ENSSSCG00000019309	SNORA63
ENSSSCG00000019937	SNORA19
ENSSSCG00000020439	RNaseP_nuc
ENSSSCG00000020672	F2R
ENSSSCG00000020770	
ENSSSCG00000020786	GNG7
ENSSSCG00000021067	BLVRB
ENSSSCG00000021119	
ENSSSCG00000021178	
ENSSSCG00000021208	
ENSSSCG00000021411	
ENSSSCG00000021443	SGK1
ENSSSCG00000021631	
ENSSSCG00000022049	HIST1H4D
ENSSSCG00000022246	
ENSSSCG00000022256	C10orf10
ENSSSCG00000023243	
ENSSSCG00000023638	

ENSSSCG00000023669	SNORD30
ENSSSCG00000024259	
ENSSSCG00000024283	
ENSSSCG00000025282	SNORA57
ENSSSCG00000025540	SNORD33
ENSSSCG00000025567	
ENSSSCG00000025631	
ENSSSCG00000025736	RECQL4
ENSSSCG00000025957	HIST1H2BH
ENSSSCG00000026214	
ENSSSCG00000026257	STMN1
ENSSSCG00000026332	
ENSSSCG00000026430	DDX3Y
ENSSSCG00000026434	
ENSSSCG00000026570	SNAI2
ENSSSCG00000026585	PROCR
ENSSSCG00000026588	
ENSSSCG00000026618	
ENSSSCG00000026923	
ENSSSCG00000027023	
ENSSSCG00000027292	
ENSSSCG00000027435	HIST1H3E
ENSSSCG00000027611	
ENSSSCG00000028244	
ENSSSCG00000028281	U2
ENSSSCG00000028439	
ENSSSCG00000028502	
ENSSSCG00000028829	HIST1H3E
ENSSSCG00000028996	
ENSSSCG00000029065	
ENSSSCG00000029264	PKMYT1
ENSSSCG00000029699	
ENSSSCG00000029747	
ENSSSCG00000029864	EIF1AY
ENSSSCG00000030023	HIST1H1B
ENSSSCG00000030143	OGN
ENSSSCG00000030157	
ENSSSCG00000030516	
ENSSSCG00000030783	SBAB-554F3.9

ENSSSCG00000030790	TMP-CH242-74M17.6
ENSSSCG00000030875	STXBP4
ENSSSCG00000030892	CU302278.1

Supplemental Table 2.

List of primers for pCR.

**PRIMER NAME**	**SEQUENCE**
COL1A1_FOR_Ss	TGCTGTTGGTGCTAAGGGTG
COL1A1_REV_Ss	GAGCACCAGCAATACCAGGA
COL3A1_FOR_Ss	GCGTCAAGGGTGAAAATGGA
COL3A1_REV_Ss	GCTGGCTGGATATTTCCTGAG
RPL32_FOR_Ss	GCAACAAATCATACTGTGCTGAGA
RPL32_REV_Ss	GCCGCTCTCTCGACGATG
BGN_FOR_Ss	TGAGTTTTCTGCCCACCCTG
BGN_REV_Ss	GAAGTCATTGACGCCCACCT
